# Unique epigenetic gene profiles define human breast cancers with poor prognosis

**DOI:** 10.18632/oncotarget.13334

**Published:** 2016-11-14

**Authors:** Samuel Peña-Llopis, Yihong Wan, Elisabeth D. Martinez

**Affiliations:** ^1^ Department of Pharmacology, UT Southwestern Medical Center, Dallas, TX, USA; ^2^ Department of Translational Oncology, National Center for Tumor Diseases and German Cancer Research Center, Heidelberg, Germany

**Keywords:** epigenetic signature, expression profiles, prognosis, molecular targets, triple negative breast cancer

## Abstract

Epigenetic enzymes are at the nexus of cellular regulatory cascades and can drive cancer-specific deregulation at all stages of the oncogenic process, yet little is known about their prognostic value in human patients. Here, we used qRT-PCR to profile at high resolution the expression of fifty-five epigenetic genes in over one hundred human breast cancer samples and patient-matched benign tissues. We correlated expression patterns with clinical and histological parameters and validated our findings in two independent large patient cohorts (TCGA and METABRIC). We found that human breast malignancies have unique epigenetic profiles and cluster into epigenetic subgroups. A subset of epigenetic genes defined an Epigenetic Signature as an independent predictor of patient survival that outperforms triple negative status and other clinical variables. Our results also suggest that breast cancer grade, but not stage, is driven by transcriptional alterations of epigenetic modifiers. Overall, this study uncovers the presence of epigenetic subtypes within human mammary malignancies and identifies tumor subgroups with specific pharmacologically targetable epigenetic susceptibilities not yet therapeutically exploited.

## INTRODUCTION

It has been estimated that epigenetic changes are ten to forty times more frequent in cancers, including breast cancer, than genetic mutations [1–4]. Recent reports have described the over-expression, amplification, fusion or mutation of many individual epigenetic enzymes across a variety of tumor types. Epigenetic enzymes have the potential to influence cellular pathways beyond control of chromatin structure [1–4], affecting the modulation of transcription factor function [5, 6] and of protein synthesis [7] and stability [8]. These facts put epigenetic enzymes at the nexus of cellular regulatory cascades and define them as potential drivers of cancer-specific deregulation at all stages of the oncogenic process.

Individual epigenetic genes have been found to be oncogenic drivers and thus therapeutic targets [9–11] or to contribute to the oncogenic process through loss of function, or new mutant activities [12, 13]. The epigenetic landscape impacts breast cancer susceptibility and affects metabolic status, oncogene addiction, tumor suppressor silencing and even the development of drug resistance [1–4, 14–19]. Here, we used quantitative high throughput RT-PCR to measure the expression of 55 epigenetic genes in over 100 fully annotated breast cancer patient samples and the corresponding patient-matched benign specimens. We then validated our results using The Cancer Genome Atlas (TCGA) data (https://tcga-data.nci.nih.gov/tcga) and, separately, using the Molecular Taxonomy of Breast Cancer International Consortium (METABRIC) dataset [20]. We found that levels of epigenetic genes distinguish normal vs. malignant tissues, that epigenetic subtypes exist within human breast cancers, and in particular, that a unique epigenetic gene signature has stronger prognostic value than triple negative status, and identifies enzymes that can be pharmacologically targeted, suggesting novel therapeutic options for human patients.

## RESULTS

### Human breast cancers exhibit altered epigenetic gene expression profiles

**Figure 1:**
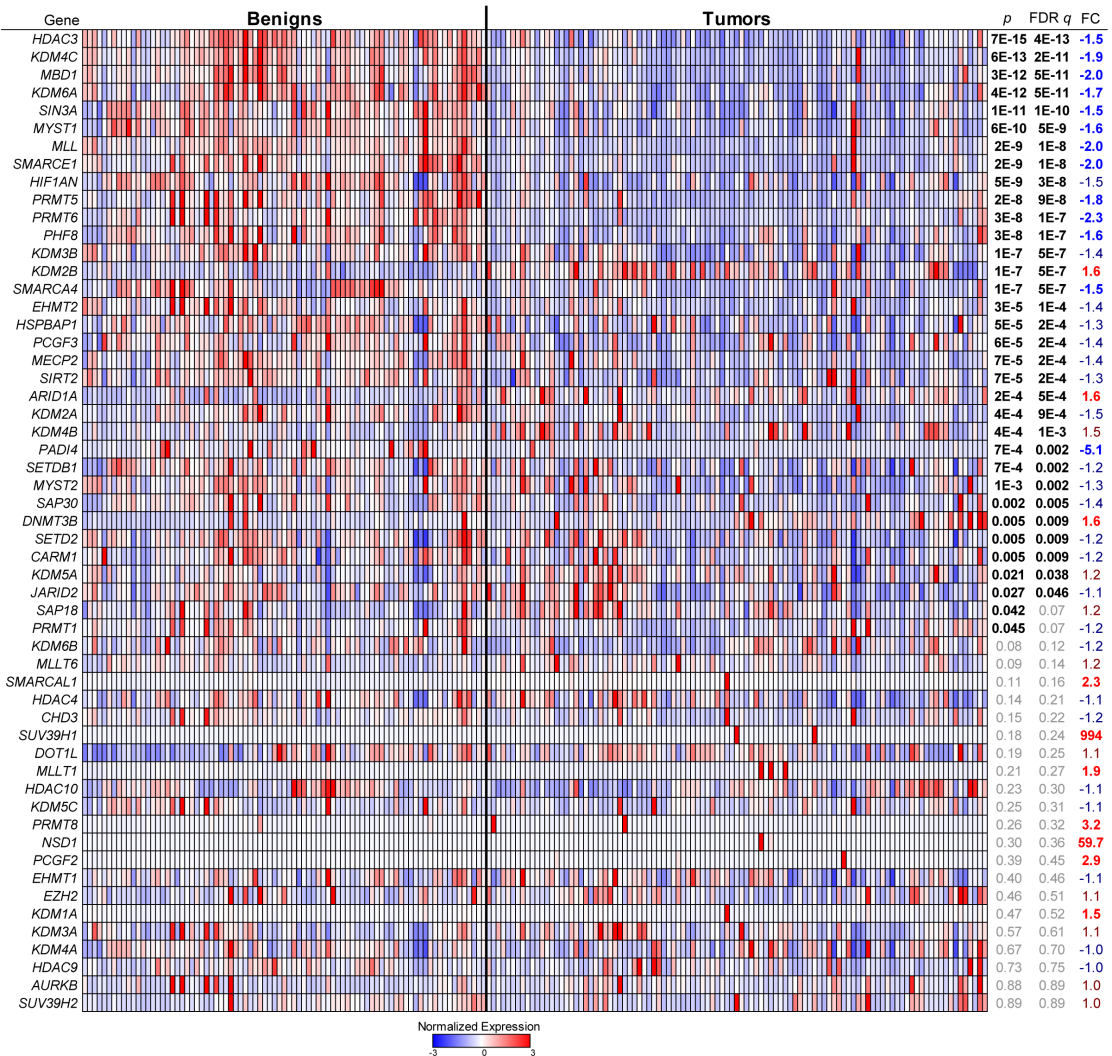
Distinct patterns of epigenetic gene expression in patient-matched benign vs. tumor tissues **A.**, Epigenetic genes were measured by qRT-PCR. For each gene, the p value, false discovery rate (FDR) q value, and fold change (FC) are given across all tumor vs. benign samples (n = 103 and 83, respectively). Expression values were categorized by the number of standard deviations away from the average (-3 to +3) for each gene across all samples and are represented in a blue to red color scale. See also Supplementary Figure 1.

We obtained over 100 tumor specimens (and more than 80 corresponding patient-matched benign tissues) through UTSTR core facility (Supplementary Table 1) scored pathologically to contain > 70% tumor tissue and otherwise randomly selected [21]. We performed qRT-PCR analysis in triplicate to measure the expression level of 55 detectable epigenetic enzyme genes (Supplementary Table 2), plus three reference and three tissue specific control genes. Expression profiles of the 55 genes were sufficient to cluster tumor samples away from benign tissue (Figure 1 and Supplementary Figure 1). No lobular breast cancers clustered with benign tissues although a few ductal samples did. Among the changes driving this benign vs. tumor separation (Figure 1 and Supplementary Figure 1A), we observed the downregulation of HDAC3 and Sirt2 as reported [22, 23]. In addition, we found altered expression of subsets of all major histone erasers, writers, readers and modifiers. For instance, among erasers, we saw general cancer downregulation of KDM4C/JMJD2C (negatively associated with invasive breast cancer [24]). In contrast, KDM2B/FBXL10 and KDM4B/JMJD2B were upregulated while KDM3A/JMJD1A, KMD2A/JMJD2A and KDM5C/JARID1C were not altered. Among histone writers, MLL, EHMT2, PRMT5 and PRMT6 were downregulated with no changes in DOT1L and MLLT6. Of note was the increased expression of ARID1A (perhaps due to mutations that decrease protein levels [25]), and of DNMT3B. Patient-matched benign vs. tumor paired analysis also revealed inter-patient heterogeneity (Supplementary Figure 1B). These results indicate that epigenetic enzyme genes are generally deregulated in breast tumors.

### Transcriptional co-regulation of subsets of epigenetic genes in patient samples

Unsupervised hierarchical clustering grouped genes into four major subsets in tumors (Supplementary Figure 2A). Jumonji enzymes are over-represented in one subset, for example, while several histone methylases cluster together with AURKB. The gene to gene correlation profiles in benign tissue followed a distinct though partly overlapping pattern compared to tumor samples (Supplementary Figure 2B). The histone methylase/AURKB cluster, for example, is not present in benign tissue. Most striking were the large distances seen between PAD14, HDAC10 or DOT1L to other genes in the benign tissue dataset, with only PADI4 remaining isolated in the tumor tissue, suggestive of new tumorigenic transcriptional networks involving HDAC10 and independently, DOT1L.

### Epigenetic gene levels define breast cancer subgroups

**Figure 2:**
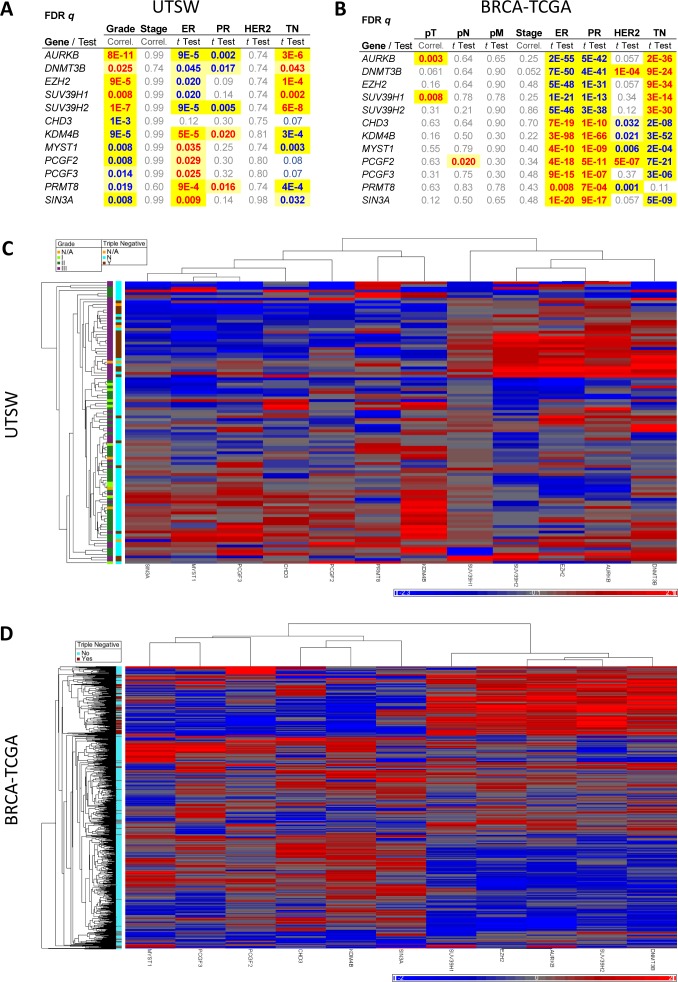
A set of epigenetic modifiers correlates with breast cancer subtypes and defines an Epigenetic Signature associated with tumor grade and triple negative status **A.**, FDR-corrected p values (q) are shown for genes showing significant associations with at least two clinical variables in our qRT-PCR-based UTSW breast cancer dataset. **B.**, FDR q values were calculated for the RNA-Seq-based BRCA-TCGA dataset analyzed for the genes of the Epigenetic Signature. In **A.** and **B.**, positive correlations are shown in red and negative correlations in blue. **C.**, Heatmap and unsupervised hierarchical clustering of tumors samples in the UTSW collection (n = 103) using the expression levels of the 12 genes shown in **A.** Grade and triple negative (TN) status of the samples is denoted by the color coded legend on the sides. **D.**, Heatmap and unsupervised hierarchical clustering of tumors samples in the BRCA-TCGA collection (n = 730) using the expression levels of the genes shown in **B.** excepting PRMT8 for which too few samples had RNA-Seq data available. Triple negative status of the samples is denoted by the color coded legend on the sides. Grade information was not available in the TCGA portal. In **C.** and **D.**, relative expression levels are shown in a blue (low) to red (high) scale representing standard deviations away from the average expression of all samples for each gene (grey). See also Supplementary Figures 3 and 4.

Upon examination of correlations between expression levels and clinical information, several new findings became evident (Figure 2A and Supplementary Figure 3). We found, for example, that even after strict false discovery rate (FDR) correction, EZH2 and AURKB levels showed robust positive correlation with triple negative (TN) malignancies and high Ki67 index, as expected from their role in tumor aggressiveness (Supplementary Figure 3A). Levels of EZH2 and AURKB increased with tumor grade and positively correlated with p53 levels. Several novel genes also positively correlated with TN disease, high Ki67 levels and tumor grade: DNMT3B, SUV39H1 and SUV39H2 (Figure 2A and Supplementary Figure 3A). Levels of several epigenetic genes negatively correlated with TN disease including KDM4B/JMJD2B, MYST1 and MYST2, PRMT8 and SIN3A. Of these, KDM4B/JMJD2B, MYST1, MYST2 and PRMT8 were specifically upregulated in grade 2 tumors, while SIN3A was downregulated in grade 3 malignancies. Additionally, CHD3 levels negatively correlated with tumor grade, and PCGF2 and PCGF3 levels positively correlated with ER+ status (Figure 2A). Altogether, twelve genes showed strong correlations with tumor grade/receptor status after stringent FDR corrections (Figure 2A), which hereafter we denominate “Epigenetic Signature”. These correlations were all lacking in benign tissue (Supplementary Figure 3B), indicating that breast cancers may fall into functional epigenetic subgroups.

To validate these findings, we analyzed the twelve genes in the Epigenetic Signature derived from our qRT-PCR UTSW cohort, in the BRCA-TCGA RNA-Seq as well as the METABRIC datasets [20] that had become publicly available. In the BRCA-TCGA set, we found that with the exception of PRMT8, for which only a few measurements were available, the remaining eleven genes showed highly significant correlations with TN tumors (Figure 2B), confirming our UTSW results. Similarly, the METABRIC dataset validated the correlation of ten of the twelve genes with TN status and additionally confirmed the correlation of nine of the Epigenetic Signature genes with tumor grade (Supplementary Figure 4A). Note that grade information is not readily available on the TCGA portal. The expression levels of the eleven validated genes (common to UTSW and either TCGA or METABRIC, or both) were sufficient to cluster tumors into therapeutically relevant subtypes including grade for UTSW and METABRIC, and TN status for UTSW, BRCA-TCGA and METABRIC (Figure 2C and 2D and Supplementary Figure 4B). Randomly selected groups of 11 genes out of 20,534 transcripts of the RNA-Seq data did not robustly segregate tumors into relevant subgroups (Supplementary Figure 4C), and neither did groups of 11 genes from the measured 55 epigenetic genes after excluding the Epigenetic Signature (Supplementary Figure 4D). To quantify the relative robustness of the separation seen with the Epigenetic Signature genes, we calculated arbitrary distances between the main cluster of normal samples (present in all cases) and the remaining samples, in TCGA. A one-sample t-test was performed to compare the distance of the eleven epigenetic genes vs. the other sets of random genes. As shown in Supplementary Figures 4C-4D, the 11 genes had a distance equal to 0 which was significantly different than the other gene sets, which gave distances ranging from 65-135 (P = 0.044, Supplementary Figure 4C; P = 0.00002 for comparison to all non-signature gene sets tested, Supplementary Figure 4C-4D).

**Figure 3:**
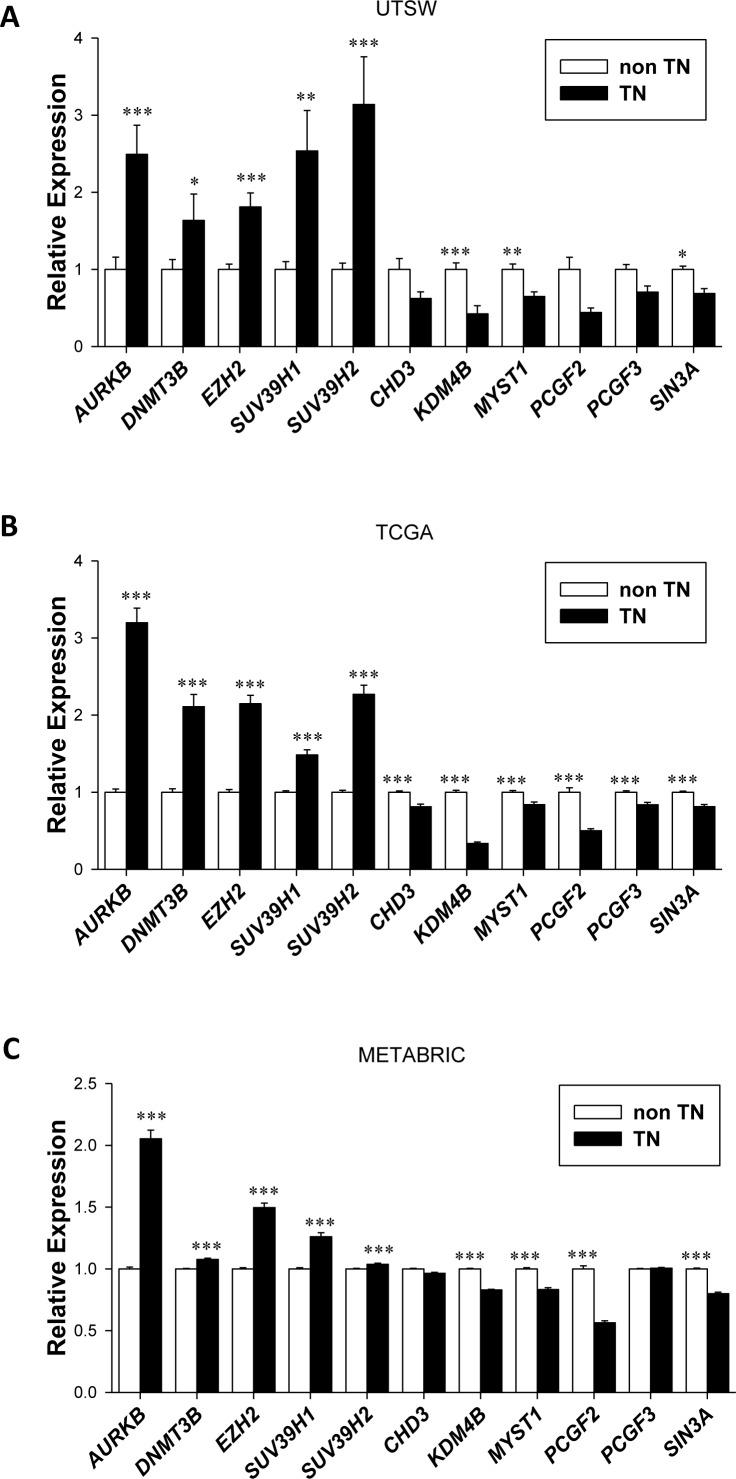
Deregulation of a subset of epigenetic modifiers in Triple Negative (TN) breast cancers across three independent datasets **A.**, Expression level in the UTSW cohort of the eleven genes from the Epigenetic Signature defined in the UTSW dataset (n = 25 TN, n = 75 nonTN) and validated in **B.** TCGA (n = 120 TN, n = 610 nonTN) and **C.** METABRIC (n = 320 TN, n = 1672 nonTN) datasets. Bars represent mean/average and error bars show standard error. Note that PRMT8 is not included due to paucity of data in TCGA for this gene.

We next examined each of the eleven genes (AURKB, DNMT3B, EZH2, SUV39H1, SUV39H2, CHD3, KDM4B, MYST1, PCGF2, PCGF3 and SIN3A) in more detail in the three datasets, and generally found significant differences in their expression in TN vs. nonTN disease (Figure 3). These differences generally also held for individual receptor status as shown in Supplementary Tables 3-4.

### The epigenetic signature is prognostic of survival

**Figure 4:**
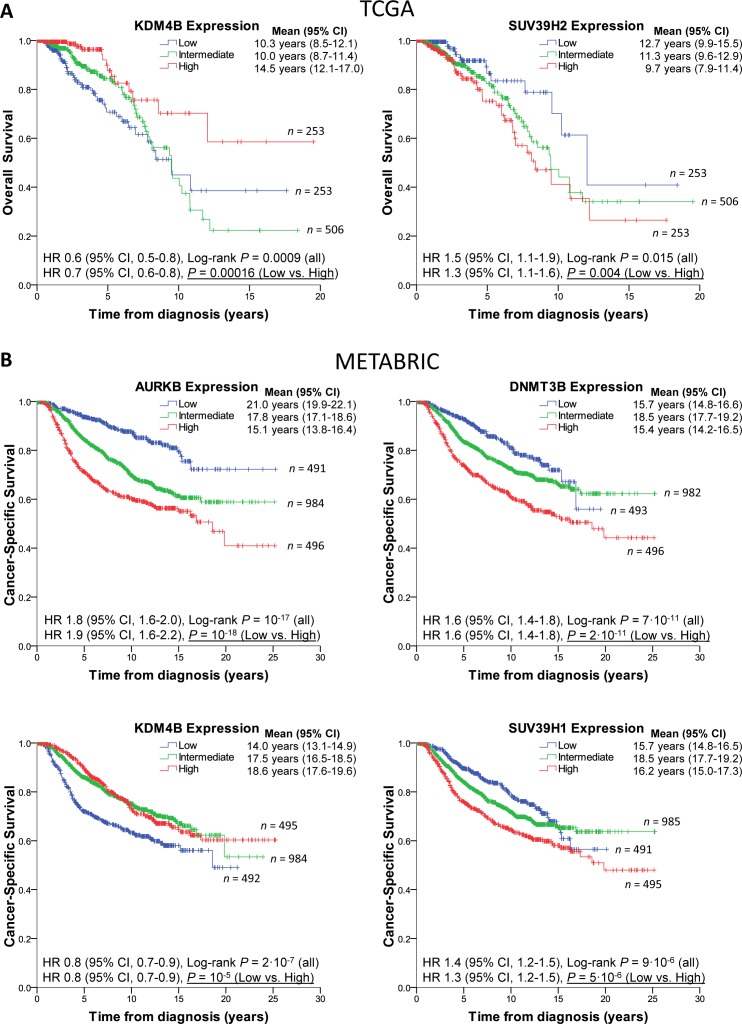
Epigenetic modifier expression levels predict breast cancer patient survival **A.**, Left: Kaplan-Meier survival curves of BRCA-TCGA patients showing low KDM4B levels (1st quartile, blue) are significantly associated with poorer survival than patients with high expression levels (4th quartile, red). Second and third quartiles are shown combined, in green. Note that KDM4B levels are negatively correlated with tumor grade and triple negative status. Right: BRCA-TCGA patients showing high levels of SUV39H2 (4th quartile, red) are significantly associated with poorer survival than patients with low expression levels (1st quartile, blue). Note that SUV39H2 levels are positively correlated with triple negative status. **B**, METABRIC patients expressing high levels of AURKB, DNMT3B or SUV39H1 (red curves) or low levels of KDM4B (blue curve) have worse prognosis. Censored cases are designated by crosses. See also Supplementary Figure 5.

To evaluate if levels of any of the eleven genes had predictive value, we examined each gene individually in the TCGA cohort, and then validated the results in METABRIC. Strikingly, levels of KDM4B on their own had prognostic value in TCGA, with patients harboring high expressing tumors surviving significantly longer (Figure 4A). SUV39H2 levels were also predictive of survival (9.7 yrs. [95% CI 7.9-11.4 yrs.] for high expressors vs. 12.7 yrs. [95% CI 9.9-15.5 yrs.] for low expressors; Figure 4A, right). Individually, the other genes in the signature did not segregate poor vs. good prognosis in TCGA. Levels of KDM4B were predictive of survival also in METABRIC as were levels of DNMT3B, SUV39H1, and as recently described [26], AURKB (Figure 4B). This larger dataset also uncovered significant prognostic value associated with PRMT8 and SIN3A (Supplementary Figure 5) as well as confirmed the well-known association of high EZH2 expression levels with poor survival in breast cancer.

**Figure 5:**
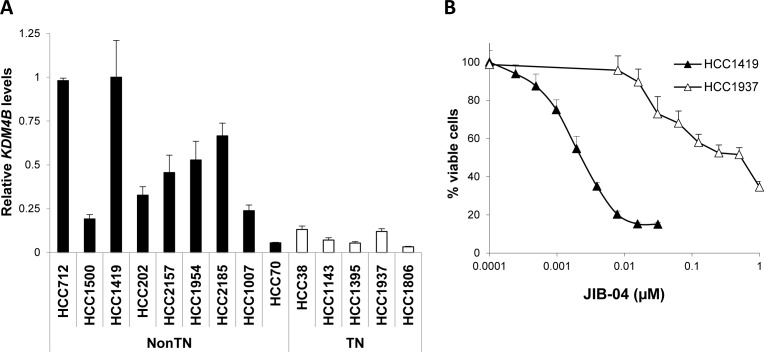
Epigenetic Signature gene KDM4B is targetable in nonTN grade 2 breast cancer cells **A.**, Levels of KDM4B measured by qRT-PCR across a panel of breast cancer cell lines based on the triple negative (TN) status is shown. Error bars represent standard deviations across triplicates. **B.**, Response of nonTN, grade 2 HCC1419 cells (high KDM4B) vs. TN, grade 3 HCC1937 cells (low KDM4B) to Jumonji inhibitor JIB-04. See also Supplementary Figure 6.

We next measured the levels of signature genes whose protein products could be pharmacologically targeted in breast cancer lines (AURKB, SUV39H1, SUV39H2 and KDM4B). KDM4B levels in cell lines correlated with TN status in agreement with patient samples (Figure 5A). We thus tested the KDM/Jumonji inhibitor JIB-04 [27] across a panel of breast cancer lines (Supplementary Figure 6) and found that the line most sensitive to JIB-04 was HCC1419, which is derived from a nonTN grade 2 tumor and expresses high levels of KDM4B (Figure 5B). The most resistant line tested of known origin, HCC1937, represents TN grade 3 disease (Figure 5B).

**Figure 6:**
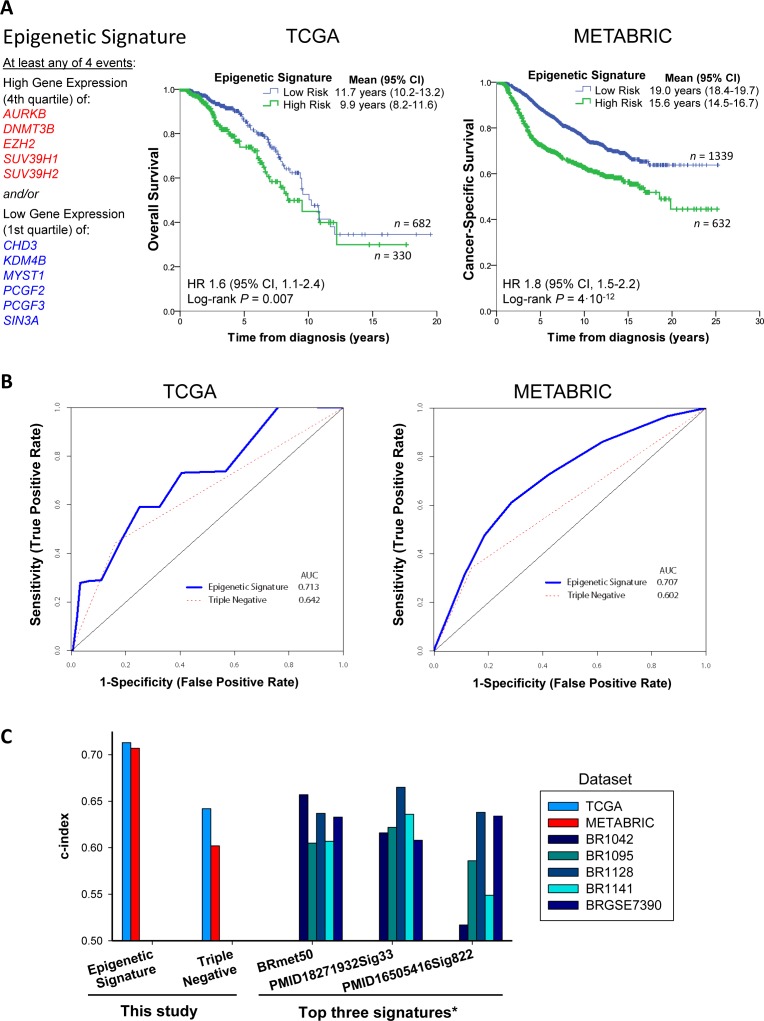
The Epigenetic Signature is a predictor of survival that outperforms other signatures **A.**, The Epigenetic Signature can identify breast cancer patients with poor survival in both TCGA and METABRIC datasets. The signature is met when at least the expression values of four positively-correlated genes with tumor grade and TN (AURKB, DNMT3B, EZH2, SUV39H1, SUV39H2) are high (in the 4th quartile of the population) and/or the expression values of four negatively-correlated genes with tumor grade and TN (CHD3, KDM4B, MYST1, PCGF2, PCGF3, SIN3A) are low (in the 1st quartile of the population). **B.**, Receiver Operator Characteristic (ROC) curve analysis of the Epigenetic Signature on patient survival using the survivalROC package in R. The area under the curve (AUC) of the Epigenetic Signature is greater than the triple negative status in the TCGA and METABRIC datasets. **C.**, Comparison of the ROC analysis of the Epigenetic Signature and TN status of this study with the data reported by Lehmann and colleagues [28] on the top three signatures in breast cancer. See also Supplementary Figure 7.

We then evaluated our Epigenetic Signature for prognostic value. We defined the Epigenetic Signature as high risk when the gene expression of at least four of the positive-correlated genes with poor prognosis (AURKB, DNMT3B, EZH2, SUV39H1, SUV39H2) are in the 4th quartile of the population and/or four of the negatively-correlated genes (CHD3, KDM4B, MYST1, PCGF2, PCGF3, SIN3A) are in the 1st quartile of the population. This criterion to meet the Epigenetic Signature is not too stringent (just one third of the Epigenetic Signature genes were sufficient to identify patients of high risk), and it gave robust prognostic value in both TCGA and METABRIC (Figure 6A), establishing epigenetic subgroups of clinical significance. Patients with high-risk Epigenetic Signature displayed poor survival in both datasets (P = 0.007 and 4·10-12, respectively; Figure 6A), with similar hazard ratios (1.6 and 1.8, respectively). The analysis of patient survival based on TN status (Supplementary Figure 7) showed lower significance in both datasets than the Epigenetic Signature (Figure 6A), indicating that the Epigenetic Signature is a stronger predictor of survival.

To further investigate the prognosis potential of the Epigenetic Signature, we performed univariate Cox regression models on the clinical variables (Table 1). Variables that were significant in the univariate Cox model were entered into an unsupervised stepwise forward conditional multivariate analysis to identify independent predictors of prognosis. The Epigenetic Signature was included in the multivariate Cox regression model of both TCGA and METABRIC (P = 2·10-4 and P = 4·10-4, respectively, Table 1), while variables that were not completely independent were not present in the final step of the model. Only high stage was a stronger independent prognostic factor in both datasets. Notably, tumor grade was an independent predictor of survival with respect to the Epigenetic Signature in METABRIC. TN status was significant in the univariate Cox model of the METABRIC dataset but excluded from the final multivariate model (Table 1), indicating that its contribution in prognosis is weaker compared to other variables, such as stage or the Epigenetic Signature. A detailed examination of the tumor characteristics of the high-risk Epigenetic Signature population revealed an over-representation of Basal-type and an under-representation of LumB and especially LumA breast cancers (Supplementary Table 5).

**Table 1:** Univariate and multivariate Cox regressions of the clinical variables and the Epigenetic Signature in the TCGA and METABRIC datasets

	TCGA						METABRIC					
	Univariate Analysis			Multivariate Analysis			Univariate Analysis			Multivariate Analysis		
Variable	HR	95% CI	P	HR	95% CI	P	HR	95% CI	P	HR	95% CI	P
Age (> median)	1.6	1.1-2.3	0.010	2.1	1.4-3.1	4·10-4	0.9	0.7-1.2	0.55			
High Grade (3 vs. 1-2)							1.8	1.5-2.2	10-10	1.4	1.1-1.8	0.003
High Stage (III-IV vs. I-II)	2.0	1.3-2.9	7·10-4	2.2	1.5-3.3	7·10-5	3.4	2.5-4.7	8·10-15	3.5	2.6-4.8	7·10-15
ER Positive	0.7	0.5-1.1	0.15				0.5	0.4-0.6	9·10-12			
PR Positive	0.7	0.5-1.0	0.042				0.5	0.4-0.6	2·10-12			
HER2 Positive	1.3	0.7-2.4	0.36				2.2	1.8-2.7	10-12	1.8	1.4-2.3	6·10-6
Triple Negative	1.7	0.9-2.9	0.08				1.5	1.2-1.9	2·10-4			
Epigenetic Signature	1.6	1.1-2.4	0.008	2.2	1.4-3.2	2·10-4	1.8	1.5-2.2	9·10-12	1.5	1.2-1.9	4·10-4

HR, Hazard Ratio; CI, Confidence Interval.

To further inspect the prognostic accuracy and diagnostic potential of the Epigenetic Signature, we performed Receiver Operating Characteristic curve analysis (Figure 6B). The Epigenetic Signature showed a greater area under the curve (AUC) than the TN status in both TCGA and METABRIC, with an average prognostic value (c-index) of 0.71 for the Epigenetic Signature and 0.62 for TN status. In addition, the c-index for the Epigenetic Signature was greater than the ones observed for the top 3 gene expression signatures out of 351 reported breast cancer signatures from 206 studies evaluated by Lehmann and colleagues [28], namely BRmet50, PMID18271932Sig33 and PMID16505416Sig822 (Figure 6C). This suggests that the Epigenetic Signature outperforms previous gene expression signatures in the prognosis of breast cancer.

## DISCUSSION

Here we have identified the epigenetic modifiers that become deregulated during human breast oncogenesis. Among these, a set of 11 epigenetic genes distinguish between TN and nonTN human breast cancer specimens and two of these genes independently offer prognostic value. Our results from this novel UTSW dataset were validated in the TCGA and METABRIC datasets confirming the presence of epigenetic subgroups within mammary malignancies and additionally showing the prognostic value of several genes from our Epigenetic Signature, in these larger cohorts. Importantly, our studies reveal that human TN disease may be targetable by inhibitors of EZH2, AURKB, DNMT3B and/or SUV39H1/2 and that nonTN tumors may respond to KDM4B and PRC1 (PCGF2/3) inhibition.

In line with reports that the estrogen receptor (ER) regulates KDM4B expression [29], we observed upregulation of KDM4B in ER+ but not in ER- or TN tumors in agreement with KDM4B's oncogenic activity in other tumor types [10, 30, 31]. In nonTN tumors we also observed upregulation of members of the PRC1 complex, PCGF2 and PCGF3 [32]. Whether this upregulation of PCGF factors defines a susceptibility to PRC1 inhibitors, such as PRT4165 [33], remains to be investigated.

In addition to the established EZH2 and the reported AURKB [34–36], three other targetable epigenetic genes were significantly upregulated in TN disease: DNMT3B, SUV39H1 and SUV39H2. The high expression of DNMT3B we report is consistent with the described hypermethylator phenotype of this breast cancer subtype [37] and itself has prognostic value (Figure 4B). Of interest, SUV39H1, shown to negatively regulate the ER promoter [38], is upregulated in TN patient samples and shows prognostic value in the large METABRIC cohort (Figure 4B). This is in agreement with the trend reported by Patani et al. in a smaller patient cohort [39] as is the better prognosis of patients with high MYST1/KAT8 (Supplementary Figure 5). The functional significance of this enzyme as well as other epigenetic modifiers in our signature (CHD3, PRMT8, SIN3A) will surely be of future interest [40–42].

Remarkably, we have identified an Epigenetic Signature that is a strong independent predictor of patient survival in the TCGA and METABRIC datasets, according to the multivariate Cox regression models. This Epigenetic Signature outperformed TN status and other clinical variables for prognosis prediction. Notably, ROC analysis revealed a c-index for the Epigenetic Signature that was greater than any of the c-indices observed for the top 3 gene expression signatures out of 351 reported breast cancer signatures from 206 studies evaluated by Lehmann and colleagues [28]. Therefore, the Epigenetic Signature has the potential to be a novel biomarker of patient survival in breast cancer. In summary, we have identified epigenetic breast cancer subgroups overall, and within TN and nonTN human breast cancers, which define novel epigenetic targets and suggest target-combinations for these malignancies.

## MATERIALS AND METHODS

### Clinical samples

The University of Texas Southwestern Medical Center Tissue Resource (UTSTR) was the source of tumor and benign samples from human patients. Samples were processed by the UTSTR after proper consent under IRB approved protocols and the first 103 samples in the collection scored to be > 70% tumor tissue for cancer samples were used for this study [21] along with matching benign tissue when available. UTSTR de-identified samples and extracted total RNA. Patient and tumor characteristics are described in Supplementary Table 1. A PAM50-like method was used to classify these breast cancers into Luminal A (LumA; tumor grade I or II, ER+, HER2- and Ki67≤18%), Luminal B (LumB; tumor grade III, ER+, HER2- and Ki67 > 18% or ER+ and HER2+), HER2 overexpressing (HER2; ER-, PR- and HER2+) and Basal/Triple Negative (TN; ER-, PR- and HER2-) [43].

### qRT-PCR

Primers sets were designed against the corresponding human genes (sequences are listed in Supplementary Table 2) and validated as previously described [44]. RNA samples from the UTSTR were quantified, DNAse treated and reverse transcribed, and the resulting complementary DNA (cDNA) was amplified in SYBR real-time quantitative PCR assays (Applied Biosystems) using a high throughput robotic platform. Reactions were performed on an ABI Prism 7900HT with an initial 2-min pre-incubation at 50°C, followed by 10 min at 95°C and then 40 cycles of 95°C for 15 sec and 60°C for 1 min. hCyclophilin was used as the reference gene and hTBP or 18S used as a second reference gene to confirm expression changes. Data were analyzed following the ∆∆ Ct method as described previously [44], using validated cDNA standard curves. Reactions were run in triplicate. Tissue specific genes were run in addition to the genes of interest and their expression patterns used in subsequent analysis to ensure correlations did not correspond to fat or stromal content of patient samples (Supplementary Figure 2). For quantification of KMD4B levels in breast cancer cell lines, RNA was extracted from exponentially growing cells and the exact same protocol and analysis was used as above except that reactions were prepared manually rather than with a robot.

### Data analysis

Gene expression data was analyzed as previously described [45]. Briefly, unpaired t tests were performed between tumor and benign samples taking into account the group variances. The gene expression for each gene was associated with clinical variables using Spearman correlations, t tests or Fisher exact tests depending on the characteristics of the variables (continuous or categorical). All calculated p values were corrected using the Benjamini and Hochberg false discovery rate (FDR) method to discard false positives by the fact of performing multiple tests. Unsupervised hierarchical clustering was computed with Partek Genomics Suite v6.6.

### TCGA data analysis

To validate the results, RNA-Seq and clinical data of breast invasive carcinoma (BRCA) was downloaded from The Cancer Genome Atlas (TCGA) data portal (https://tcga-data.nci.nih.gov/tcga) on June 14th, 2014 and tested for associations as indicated [46]. Gene expression levels were estimated by the RNA-Seq Expectation-Maximization (RSEM) normalization method and analyzed as described above. The median and quartiles for the expression of each gene was calculated for all available patients. Gene expression was considered low for patients with expression values in the 1st quartile, intermediate for the 2nd and 3rd quartiles and high for the 4th quartile. Tumors with negative estrogen receptor (ER) status, progesterone receptor (PR) status, and HER2 status by either IHC or FISH were considered as triple negatives. Tumors with undetermined or not evaluated status for any of ER, PR or HER2 were excluded. Overall survival was calculated from the date of diagnosis to the date on which the patient dies from any cause. Patients alive at the end of the study period were censored at the date of last follow-up or the last date the patient was known to be alive, whichever was longer. Kaplan-Meier survival curves, log-rank tests and Cox regression models were calculated with SPSS Statistics 17. The hazard ratio (HR) and the 95% confidence intervals (CI) were estimated for each variable using univariate Cox regression models. To identify independent predictors of survival, only the significant variables in the univariate Cox regression were entered into the multivariate Cox proportional hazard model using a forward conditional method considering the default stepwise probabilities of 0.05 for entry and 0.10 for removal of covariates from the model.

### METABRIC data analysis

Clinical information from the Molecular Taxonomy of Breast Cancer International Consortium (METABRIC) was obtained from the original publication [20]. ER, PR and HER2 status was considered based on their reported expression. Gene expression data from METABRIC using the Illumina HT-12 platform was downloaded from the European Genome-Phenome Archive (http://www.ebi.ac.uk/ega/) under accession number EGAS00000000083. Gene expression values for probes corresponding to the same gene were pooled. Only cancer-specific survival was considered and analyzed as described above.

### ROC analysis

Receiver Operating Characteristic (ROC) curve analysis for the Epigenetic Signature and triple negative status on patient survival was performed using the survivalROC package in R 3.2.5 as described [47]. For the Epigenetic Signature, the total amount of genes in the 1st and 4th quartile, according to the signature definition (Figure 6C), were taken into account for each patient instead of considering them only as low or high risk. Half of the median survival was considered as the time point of interest. The Area Under the Curve (AUC) was computed with the Kaplan-Meier estimator.

### Cell culture and viability assays

Breast cancer cell lines were the gift of Dr. D. Euhus and Dr. J. Minna and include the following lines established at our institution: HCC712, HCC1500, HCC1419, HCC202, HCC2157, HCC1954, HCC2185, HCC1007, HCC70, HCC38, HCC1143, HCC1395, HCC1937 and HCC1806. Cell lines were previously characterized as described [48] and were routinely fingerprinted and mycoplasma tested, grown in RPMI media supplemented with 5-10% fetal bovine serum.

For cell viability assays, cells were plated at low density in 96-well plates and grown overnight, then exposed to increasing doses of drug treatment or vehicle control. Standard MTS viability assays were performed on the 4th day of treatment and IC_50_ calculated, as described [27]. JIB-04 was synthesized in-house, as previously reported [27].

## SUPPLEMENTARY MATERIALS FIGURES AND TABLES




