# Supplementary material for: Unique epigenetic gene profiles define human breast cancers with poor prognosis
Source: Oncotarget. 2016 Nov 14;7(52):85819–31. doi: 10.18632/oncotarget.13334 (PMC5349877; doi:10.18632/oncotarget.13334)
Supplement: Supplementary file 1 [file oncotarget-07-85819-s001.pdf]

Supplementary Table 1. Patient and tumor information for the UTSW cohort of breast cancers.

Age	
Median	56
Race	
Caucasian	72
African American	18
Other	3
Grade	
I	9
II	42
III	48
Stage	
I	17
II	51
III	29
IV	6
ER Positive	
No	34
Yes	64
PR Positive	
No	47
Yes	51
HER2 Positive	
No	79
Yes	18
Triple Negative	
No	74
Yes	25
PAM50-like	
Triple Negative	25
HER2	9
LumA	26
LumB	33
Ploidy	
Aneuploid	52
Diploid	26
Multiploid	15
p53 Positive	
No	62
Yes	31
Ki67 Positive	
No	23
Yes	70

Data are number of patients except for Age (years).

Supplementary Table 2. qRT-PCR primers used.

Reference Genes:

Primer Name	Accession#	Forward Sequence	Reverse Sequence
18S	X00686, M10098	5'-ACCGCAGCTAGGAATAATGGA-3'	5'-GCCTCAGTTCCGAAAACCA-3'
hCYCLOPHILIN	NM_000942	5'-GGAGATGGCACAGGAGGAA-3'	5'-GCCCGTAGTGCTTCAGTTT-3'
hTBP	M55654	5'-CTTCCGCTGGCCCATAGT-3'	5'-ACGCCAAGAAACAGTGATGCT-3'

Genes of Interest:

Primer Name	Accession#	Forward Sequence	Reverse Sequence
hAOF2	NM_015013	5'-CTAATGCCACACCTCTCTCAACTC-3'	5'-CTAATGCCACACCTCTCTCAACTC-3'
haP2 *	NM_001442.2	5'-TGACGAAGTCACTGCAGATGA -3'	5'-AGGACACCCCCATCTAAGGTTA-3'
hARID1A	NM_139135.2	5'-CGATGGGACCACGACAGC-3'	5'-GGCCCTATTCCAGGCTCC-3'
hAUKRB	NM_004217.2	5'-GCAGAAGAGCTGCACATTTGAC-3'	5'-TCTGCCAACTCCTCCATGATC-3'
hCARM1	NM_199141.1	5'-GCAACAGCGTCCTCATCCA-3'	5'-GCAGGTTTTCAGGATGTTGTAGAA-3'
hCHD3	NM_005852.3	5'-GCTGTGTCCCCGATGCA-3'	5'-CACCGCCAATGTAGGATCTTC-3'
hDNMT3B	NM_175849.1	5'-CATGAAGGTTGGCGACAAGA-3'	5'-GCATCAATCATCACTGGATTACACT-3'
hDOT1L	NM_032482.2	5'-GAGTGGAGGGAGCGAATCG-3'	5'-GATCCACCTCAGGACCAAAGG-3'
hEHMT1	NM_024757.3	5'-GGAGGAACTGCCGAAATCG-3'	5'-GTCCCGCGTCCGGTAGA-3'
hEHMT2	NM_025256.5	5'-TGATGTGAGAGAGGATGATTCTTACC-3'	5'-GCATCTATGCAGTACACCTCTCCAT-3'
hEZH2	NM_152998.11	5'-GCTTTTCTGTAGGCGATGTTTTAAA-3'	5'-CCGCTTATAAGTGTTGGGTGTTG-3'
hFBXL10	NM_001005366	5'-GCGCTCCCACCTCACTCA-3‘	5'-CCGAAGAGAAGCCGTCTATGC-3'
hFBXL11	NM_012308.1	5'-TCCACCGGCTGATAAACCA-3‘	5'-AGCCGGAAGTCGGTCATGT-3'
hHDAC3	NM_003883	5'-GCCTTCAACGTAGGCGATGA-3'	5'-TAACGCGAGCAGAACTCAAAGA-3'
hHDAC4	NM_006037.3	5'-AATCTGAACCACTGCATTTCCA-3'	5'-GAGGTCGACACTCCGCTCTG-3'
hHDAC9	NM_058176	5'-AGCGAATGTTTGAGGTGACAGA-3'	5'-CAGTTGGCCCATTGTTTGGT-3'
hHDAC10	NM_032019.5	5'-GCTTCACTGTCAACCTGCCC-3'	5'-AGTCAGCGTTTCCCATCCC-3'
hHIF1AN	NM_017902.2	5'-GGCCCTGGTGATGTTCTTTACA-3'	5'-TGATGGTAATCCCCCCATTTAG-3'
hHSPBAP1	NM_024610.4	5'-TCAAAGACATGCGGTTACACTGA-3'	5'-TGACAGTGACAGGATCAATGGATT-3'
hJARID1A	NM_005056.2	5'-TGTGTTGAGCCAGCGTATGG-3'	5'-CCACCCGGTTAAAAGCAGACT-3'
hJARID2	NM_004973.2	5'-TGTTCACAACGGGCATGTTT-3'	5'-TTGTGTTTTTGAACAGGTTCCTTCT-3'
hJMJD1A	NM_018433.4	5'-GTGGTTTTCAGCAACCGTTATAAA-3'	5'-CAGTGACGGATCAACAATTTTCA-3'
hJMJD1B	NM_016604.3	5'-TGCCCTTGTATCAGTCGACAGA-3'	5'-GCACTAGGGTTTATGCTAGGAAGCT-3'
hJMJD2A	NM_014663.2	5'-TGCAGATGTGAATGGTACCCTCTA-3'	5'-CACCAAGTCCAGGATTGTTCTCA-3'
hJMJD2B	NM_015015.2	5'-GGCCTCTTCACGCAGTACAATAT-3'	5'-CCAGTATTTGCGTTCAAGGTCAT-3'
hJMJD2C	NM_015061.2	5'-GAATGCTGTCTCTGCAATTTGAGA-3'	5'-CAACGGCGCACATGACAT-3'
hJMJD3	NM_001080424	5'-CGGAGACACGGGTGATGATT-3'	5'-CAGTCCTTTCACAGCCAATTCC-3'
hKDM5C	NM_004187.3	5'-GAGGAGGGCTCAGGTAAGAGAGA-3'	5'-TGGCAACAGCGAGGACAG-3'
hMBD1	NM_015847	5'-GCGCCGCGAAGTCTTTC-3'	5'-TCGAGTCAGCTCAACTTTGCTT-3'
hMECP2	NM_00110792.1	5'-TGGGAAGTATGATGTGTATTTGATCA-3‘	5'-TGTCGCCTACCTTTTCGAAGTAC-3'
hMLL	NM_005933.2	5'-CTTCTAAGGAGGCAGTTGGTGTCTAC-3‘	5'-GACCCCGGCCATGGAT-3'
hMLLT1	NM_005934.2	5'-GGATGAGCTGGTGGAGCTACAC-3'	5'-CAGTCTCCTCGATCAGATTCACA-3'
hMLLT6	NM_005937.3	5'-CGCCATGGATGTCGACAAG-3'	5'-TGTGCCGGGATGTCTTCA-3'
hMYST1	NM_032188	5'-GCCGGCGACCGGATAG-3'	5'-CGTTCACTCGAGACTGGATCAC-3'
hMYST2	NM_007067	5'-TCAGGATGCCCACTGTATCATAAC-3'	5'-ATCTGCTTATCCCGGCTCTGT-3'
hNSD 1	NM_172349.2	5'-CCCGAGTCTTCCCTTACATGG-3'	5'-GCTGTCTTAGCTCTTTTTGGGC-3'
hPADI4	NM_012387.1	5'-TGGGCACCTTGACTCAGCTT-3'	5'-GAGGCGTTGATGCTGAAGGA-3'
hPCGF2	NM_007144.2	5'-GCCGCTGCTGAGCATCA-3'	5'-AAAAGCCCAGGGACCAATTT-3'
hPCGF3	NM_006315.4	5'-GGAGGTGCCGGGAGACAT-3'	5'-CTGTCGTCTGCTTTGGTTTCAC-3'
hPHF8	NM_015107.2	5'-CCTCTGTCTGGTACCATGTACTCAA-3'	5'-TCCAGCACTCAAAGAGAGTCAGAT-3'
hPRMT1	NM_198318.2	5'-CCGGCAGTACAAAGACTACAAGATC-3'	5'-GCAAGACATGTCGAAGCCATAC-3'
hPRMT5	NM_00103961	5'-CCAGCCCTGTTTCACCTTCA-3'	5'-GCAATAGCGGTTGTTGTCAATC-3'
hPRMT6	NM_018137	5'-TGCTGCTGCGCTACAAAGTG-3'	5'-GCTCAGTCCTCCATGGCAAA-3'
hPRMT8	NM_109854	5'-CCAAAGCAAGTGGTGACCAA-3'	5'-AGCTCTTCCGTCTTCACTGTGTAA-3'
hSAP18	NM_005870.3	5'-GGAAATTAAGAAGGAGCCAGAGAA-3'	5'-CGTAGCAACAGTGGGCATGT-3'
hSAP30	NM_003864.3	5'-GAGTGATGATGATGGAGGTGATTC-3'	5'-TGGTTGGTAGCTTGAAGTGTCTTT-3'
hSCGB2A2*	NM_003357	5'-ACTCTGAGCAATGTTGAGGTGTTT-3'	5'-CCAAAGGTCTTGCAGAAAGTTAAAA-3'
hSETD2	NM_014159.6	5'-AAAATGGACTGTGAACGGACAA-3'	5'-TGGCTGATCCGCAGAAACA-3'
hSETDB1	NM_012432.2	5'-TGAGACACCAAACGTCAAAAACA -3'	5'-ACATAGGAAGCATAGCCATCATCA-3'
hSIN3A	NM_015477.2	5'-CTGAGCTTCGTGAACATCTAGCA-3'	5'-CTCTCGACCACGTTGACACTTC-3'
hSIRT2	NM_030593.1	5'-AGCTGCCGGCACGAATAC-3'	5'-TTGGGCGTCACCTCAGAGA-3'
hSMARCA4	NM_001128845	5'-GCAGCCTCAACGACCTAGAGA-3'	5'-GGTGAAGACCGACTGCAAGAC-3'
hSMARCAL1	NM_014140.3	5'-AGGTGGTCCTGGACGCAAT-3'	5'-AGCCATCGATGCGGATGT-3'
hSMARCE1	NM_003079.4	5'- GCCGCTGATGCCCTACAT-3'	5'-TAGGTCAGGGTTGGAAGCCTTT-3'
hSUV39H1	NM_003173	5'-AGGCCCGAATGTCGTTAGC-3'	5'-GCTGATTCCAAGAAGACTTGCA-3'
hSUV39H2	NM_024670	5'-CGGCAATGTGTCTCATTTTGTG-3'	5'-AGACGAGTATCGAGGTTATCAATGAA-3'
hUTX	NM_021140.2	5'-CACAGTACCAGGCCTCCTCATT-3'	5'-TCACTATCTGAGTGGTCTTTATGATGACT-3'

* tissue-specific genes

Supplementary Table 3. Relative Expression of the eleven genes of the Epigenetic Signature in the TCGA cohort.

		ER				PR				HER2				Triple Neg		
		n	Mean ± SE			*n*	Mean ± SE			*n*	Mean ± SE			*n*	Mean ± SE	
AURKB	-	221	517 ± 25		-	315	428 ± 20		-	567	258 ± 12		N	610	186 ± 7	
	+	746	180 ± 6	***	+	649	176 ± 7	***	+	168	238 ± 16		Y	120	595 ± 35	***
CHD3	-	221	3353 ± 99		-	315	3710 ± 105		-	567	4165 ± 71		N	610	4215 ± 68	
	+	746	4376 ± 66	***	+	649	4358 ± 67	***	+	168	3847 ± 125		Y	120	3419 ± 140	***
DNMT3B	-	221	338 ± 19		-	315	298 ± 15		-	567	167 ± 8		N	610	152 ± 7	
	+	746	137 ± 5	***	+	649	128 ± 5	***	+	168	224 ± 17	*	Y	120	321 ± 24	***
EZH2	-	221	836 ± 38		-	315	737 ± 31		-	567	490 ± 17		N	610	416 ± 14	
	+	746	397 ± 10	***	+	649	380 ± 9	***	+	168	511 ± 36		Y	120	892 ± 46	***
KDM4B	-	221	1201 ± 68		-	315	1617 ± 81		-	567	2969 ± 84		N	610	3219 ± 79	
	+	746	3430 ± 74	***	+	649	3546 ± 79	***	+	168	2525 ± 143	*	Y	120	1081 ± 60	***
MYST1	-	221	602 ± 19		-	315	619 ± 15		-	567	722 ± 15		N	610	723 ± 14	
	+	746	746 ± 13	***	+	649	758 ± 15	***	+	168	640 ± 24	**	Y	120	606 ± 25	***
PCGF2	-	221	949 ± 84		-	315	1176 ± 96		-	567	1041 ± 23		N	610	1462 ± 84	
	+	746	1405 ± 66	***	+	649	1365 ± 67		+	168	2334 ± 286	***	Y	120	730 ± 39	***
PCGF3	-	221	1235 ± 32		-	315	1341 ± 30		-	567	1468 ± 24		N	610	1506 ± 27	
	+	746	1535 ± 23	***	+	649	1528 ± 25	***	+	168	1455 ± 63		Y	120	1260 ± 46	***
SIN3A	-	221	1783 ± 42		-	315	1923 ± 48		-	567	2201 ± 32		N	610	2231 ± 30	
	+	746	2289 ± 26	***	+	649	2291 ± 25	***	+	168	2061 ± 48		Y	120	1813 ± 61	***
SUV39H1	-	221	421 ± 15		-	315	373 ± 10		-	567	315 ± 6		N	610	293 ± 5	
	+	746	286 ± 4	***	+	649	290 ± 5	***	+	168	326 ± 13		Y	120	435 ± 19	***
SUV39H2	-	221	459 ± 19		-	315	389 ± 15		-	567	259 ± 8		N	610	213 ± 5	
	+	746	202 ± 4	***	+	649	198 ± 4	***	+	168	257 ± 11		Y	120	484 ± 25	***

*, FDR q<0.05
**, FDR q<0.01
***, FDR q<0.001

Supplementary Table 4. Relative Expression of the twelve genes of the Epigenetic Signature in the METABRIC cohort.

	ER				PR				HER2				Triple Neg			
		n	Mean ± SE			*n*	Mean ± SE			*n*	Mean ± SE			*n*	Mean ± SE	
AURKB	-	474	244 ± 7	***	-	943	191 ± 4	***	-	1743	151 ± 3	***	N	1672	132 ± 2	***
	+	1518	126 ± 2		+	1049	122 ± 2		+	249	181 ± 5		Y	320	271 ± 9	
CHD3	-	474	55.8 ± 0.4		-	943	56.2 ± 0.3		-	1743	56.5 ± 0.2		N	1672	56.4 ± 0.2	
	+	1518	56.5 ± 0.2		+	1049	56.5 ± 0.3		+	249	55.6 ± 0.5		Y	320	56.3 ± 0.5	
DNMT3B	-	474	57.7 ± 0.5	***	-	943	55.2 ± 0.3	***	-	1743	51.9 ± 0.2	***	N	1672	52.2 ± 0.2	***
	+	1518	51.3 ± 0.1		+	1049	50.7 ± 0.2		+	249	59.1 ± 0.6		Y	320	56.1 ± 0.5	
EZH2	-	474	116 ± 2	***	-	943	100 ± 1	***	-	1743	86.8 ± 0.9	***	N	1672	82 ± 1	***
	+	1518	80 ± 1		+	1049	78 ± 1		+	249	100 ± 2		Y	320	122 ± 3	
KDM4B	-	474	45.3 ± 0.2	***	-	943	49.2 ± 0.3	***	-	1743	53.3 ± 0.2	***	N	1672	54.1 ± 0.2	***
	+	1518	54.9 ± 0.3		+	1049	55.7 ± 0.3		+	249	47.8 ± 0.4		Y	320	44.9 ± 0.3	
MYST1	-	474	258 ± 4	***	-	943	279 ± 3	***	-	1743	315 ± 3	***	N	1672	316 ± 3	***
	+	1518	323 ± 3		+	1049	334 ± 4		+	249	258 ± 5		Y	320	263 ± 5	
PCGF2	-	474	363 ± 24		-	943	387 ± 16	*	-	1743	313 ± 3	***	N	1672	395 ± 10	***
	+	1518	369 ± 8		+	1049	351 ± 7		+	249	750 ± 60		Y	320	223 ± 6	
PCGF3	-	474	46 ± 0.2	*	-	943	45.7 ± 0.2	*	-	1743	45.4 ± 0.1	*	N	1672	45.4 ± 0.1	
	+	1518	45.3 ± 0.1		+	1049	45.3 ± 0.1		+	249	46.1 ± 0.3		Y	320	45.7 ± 0.3	
PRMT8	-	474	42 ± 0.2	*	-	943	42.1 ± 0.2	***	-	1743	42.7 ± 0.2	**	N	1672	42.8 ± 0.2	*
	+	1518	42.8 ± 0.2		+	1049	43.1 ± 0.2		+	249	42 ± 0.2		Y	320	41.9 ± 0.3	
SIN3A	-	474	391 ± 5	***	-	943	435 ± 4	***	-	1743	471 ± 3	***	N	1672	482 ± 3	***
	+	1518	490 ± 3		+	1049	495 ± 4		+	249	432 ± 9		Y	320	385 ± 6	
SUV39H1	-	474	305 ± 6	***	-	943	278 ± 4	***	-	1743	251 ± 2	***	N	1672	246 ± 2	***
	+	1518	240 ± 2		+	1049	236 ± 2		+	249	290 ± 7		Y	320	310 ± 8	
SUV39H2	-	474	66.1 ± 0.5	***	-	943	65.3 ± 0.3	***	-	1743	63.9 ± 0.2		N	1672	63.6 ± 0.3	***
	+	1518	63.4 ± 0.3		+	1049	62.8 ± 0.3		+	249	64.9 ± 0.7		Y	320	66 ± 0.5	

*, FDR $q<0.05$
**, FDR $q<0.01$
***, FDR $q<0.001$

Supplementary Table 5. Tumor characteristics of the Epigenetic Signature.

	UTSW			TCGA			METABRIC		
	Epigenetic Signature			Epigenetic Signature			Epigenetic Signature		
Variable	Low Risk	High Risk	*P*	Low Risk	High Risk	*P*	Low Risk	High Risk	*P*
<u>Grade</u>									
I	7	2	**$3\cdot10^{-7}$**				158	12	**$3\cdot10^{-74}$**
II	36	6					659	116	
III	16	32					462	495	
<u>Stage</u>									
0			0.45			**0.006**	338	154	**0.003**
I	13	4		127	42		279	93	
II	28	23		364	210		385	194	
III	17	12		163	65		55	35	
IV	4	2		9	8		10	0	
<u>ER Positive</u>									
No	10	24	**$4\cdot10^{-6}$**	30	191	**$2\cdot10^{-14}$**	119	355	**$4\cdot10^{-14}$**
Yes	50	14		622	124		1236	282	
<u>PR Positive</u>									
No	22	25	**0.007**	97	218	**10^{-14}**	464	479	**$2\cdot10^{-14}$**
Yes	38	13		553	96		891	158	
<u>HER2 Positive</u>									
No	47	32	0.79	393	174	**0.020**	1243	500	**$9\cdot10^{-15}$**
Yes	12	6		100	68		112	137	
<u>Triple Negative</u>									
No	57	17	**$9\cdot10^{-8}$**	480	130	**$2\cdot10^{-14}$**	1289	383	**$2\cdot10^{-14}$**
Yes	4	21		11	109		66	254	
<u>PAM50</u>									
Basal*	4	21	**10^{-6}**	5	92	**$5\cdot10^{-71}$**	57	274	**$8\cdot10^{-170}$**
HER2	6	3		22	36		87	153	
LumA	22	4		225	6		686	35	
LumB	24	9		89	38		339	153	
Normal							181	21	

***:** Basal-like for the UTSW cohort corresponds to triple negatives, as indicated in the Methods. Fisher exact test was used to calculate *p* values.

A

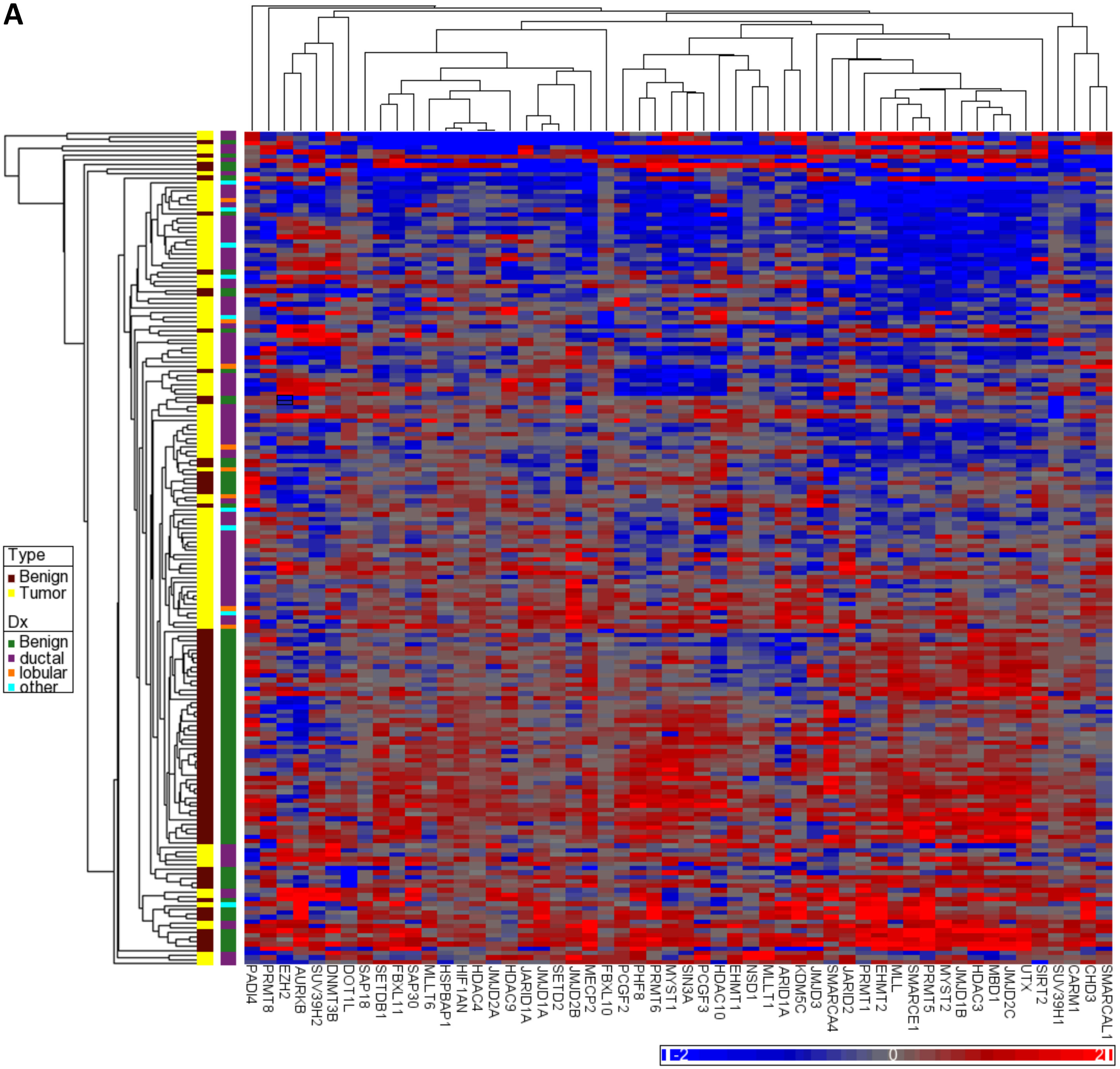

Supplementary Figure 1. *Unsupervised hierarchical clustering of epigenetic enzyme expression generally groups tumors away from benign samples.* Epigenetic enzymes in the tumors (*n*=103) and benign tissues (*n*=83) of the UTSW cohort were measured by qRT-PCR and log2-transformed levels of expression were used for clustering analysis. Expression values were categorized by the number of standard deviations away from the average (-2 to +2) for each gene across all samples, and are represented in a blue to red color scale. Tissue type (benign vs. tumor) is shown on a brown/yellow side scale and histological diagnosis (Dx) is shown on a separate color scheme as indicated in the side legend. **B**, Epigenetic enzymes (labeled on the top x axis) were measured by qRT-PCR across a panel of human breast tumors (left, *n*=103) and corresponding patient benign tissues when available (middle, *n*=72). Expression values were categorized by the number of standard deviations away from the average (-3 to +3) for each gene across tumors or across benign tissues and are represented in a blue to red color scale. The right panel shows the expression ratio of tumor to patient-matched benign.

S1B

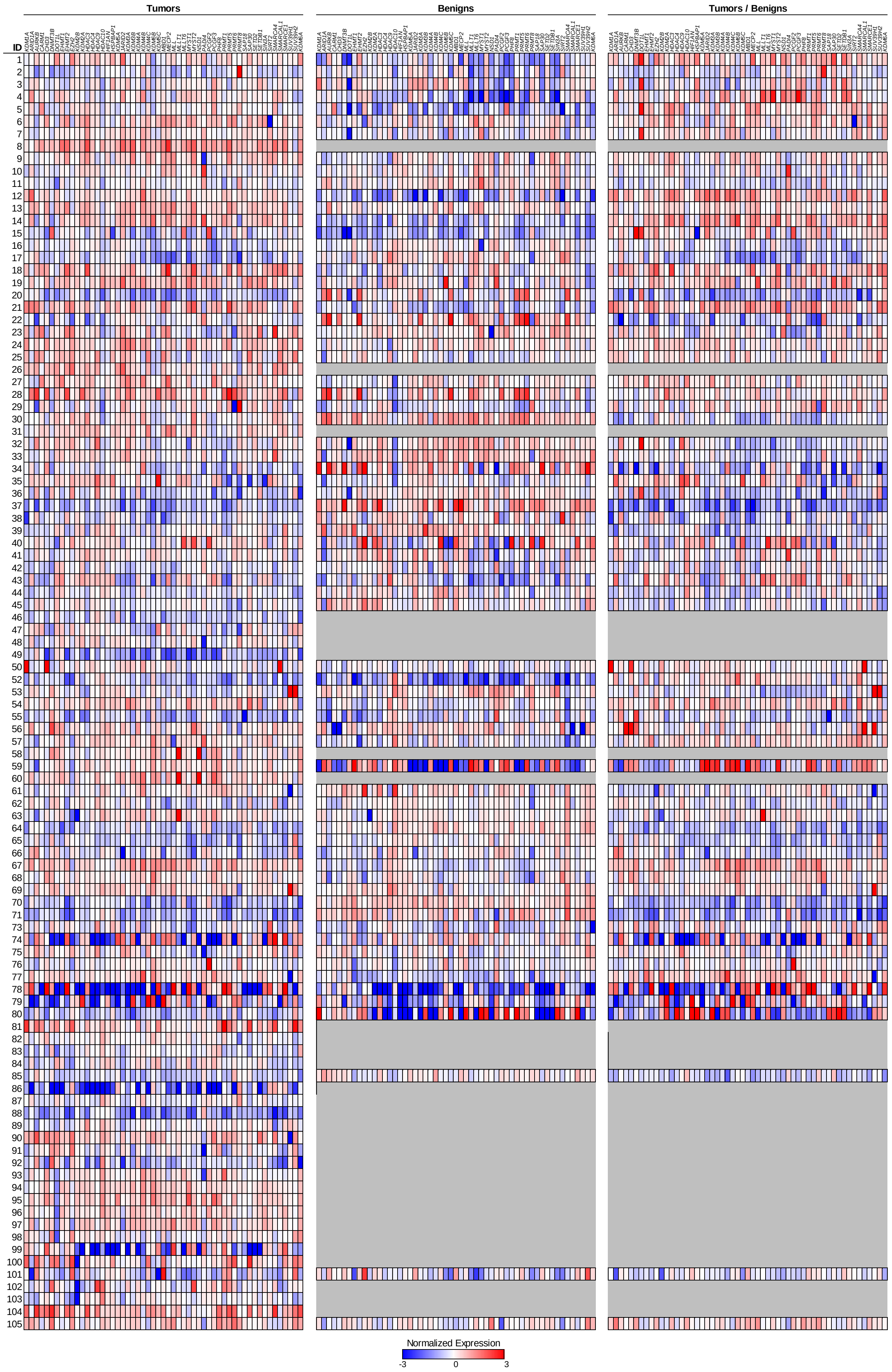
Tumors
Benigns
Tumors / Benigns
ID
Normalized Expression
-3
0
3

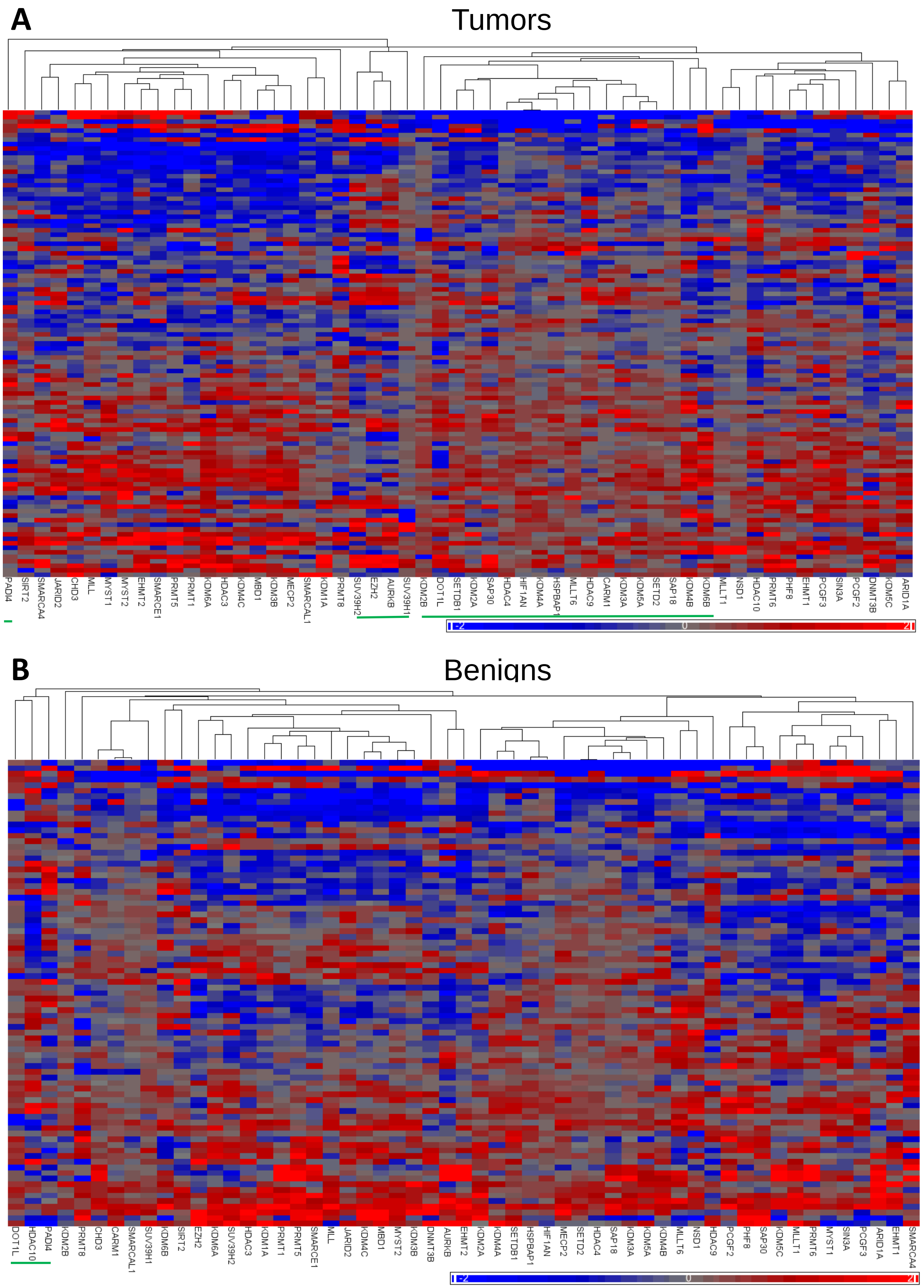

Supplementary Figure 2. *Differential co-expression of epigenetic modifiers in tumor vs. benign breast tissue*. **A**, Unsupervised hierarchical clustering of UTSW tumor samples (*n*=103) based on log2-transformed expression levels categorizes tumors into 5 major subgroups characterized by co-expression of epigenetic modifiers. **B**, Co-expression of epigenetic modifiers in UTSW patient benign breast tissue (*n*=83) follows a pattern distinct from tumor tissue, helping define cancer-specific new epigenetic transcriptional networks. Expression values were categorized by the number of standard deviations away from the average (-2 to +2) for each gene across all samples, and are represented in a blue to red color scale.

A

UTSW Tumors

FDR *q*

Gene / Test	Age Corr.	Race Fisher	Grade Corr.	Stage Corr.	ER Corr.	ER *t* Test	PR Corr.	PR *t* Test	HER2 IHC Corr.	HER2 FISH Corr.	HER2 *t* Test	TN *t* Test	Ploidy Fisher	p53 *t* Test	Ki67 Corr.	PrevTx *t* Test	PostTx *t* Test	Status *t* Test
ARID1A	0.97	0.89	0.20	0.99	0.64	0.88	0.84	0.93	0.71	0.88	0.74	0.72	1.00	0.34	0.69	0.87	0.94	0.09
AURKB	0.96	0.89	**8E-11**	0.99	**0.008**	**9E-5**	**1E-4**	**0.002**	0.62	0.60	0.74	**3E-6**	0.21	**0.013**	**1E-13**	1.00	0.94	0.63
CARM1	0.96	0.76	0.81	0.99	0.95	0.86	0.50	0.99	0.96	0.75	0.74	0.33	0.55	0.70	0.14	0.88	0.94	0.87
CHD3	0.96	0.89	**1E-3**	0.99	**0.046**	0.12	**0.038**	0.30	0.63	0.66	0.75	0.07	0.80	0.09	0.14	1.00	0.94	0.63
DNMT3B	0.98	0.97	**0.025**	0.74	0.28	**0.045**	**0.001**	**0.017**	0.86	0.57	0.74	**0.043**	0.63	0.87	**5E-4**	0.88	0.96	0.96
DOT1L	0.96	0.29	0.60	0.99	0.53	0.75	0.36	0.50	0.45	0.50	0.74	0.09	1.00	0.99	0.06	0.88	0.96	0.96
EHMT1	0.96	0.69	0.06	0.99	0.05	0.12	0.31	0.86	0.55	0.75	0.75	0.49	0.80	0.81	0.10	0.91	0.94	0.96
EHMT2	0.96	0.89	0.55	0.99	0.78	0.37	0.54	0.42	0.93	0.76	0.84	0.56	0.43	0.94	0.06	0.92	0.96	0.65
EZH2	0.96	0.94	**9E-5**	0.99	0.06	**0.020**	0.06	0.09	0.45	0.46	0.74	**1E-4**	0.21	**0.003**	**4E-8**	0.88	0.94	0.28
HDAC10	0.96	0.59	0.86	**0.038**	0.48	0.40	0.80	0.87	0.41	0.66	0.89	0.63	0.55	0.81	0.34	0.88	0.94	0.71
HDAC3	0.96	0.59	0.32	0.99	0.75	0.88	0.90	0.87	0.93	0.66	0.75	0.84	0.55	0.64	0.84	0.88	0.96	0.73
HDAC4	0.96	0.38	0.09	0.99	0.28	0.73	0.16	0.99	0.93	0.92	0.74	0.58	0.95	1.00	0.21	0.87	0.96	0.47
HDAC9	0.96	0.65	0.19	0.99	0.54	0.51	0.06	0.13	0.62	0.75	0.74	0.07	0.55	0.94	0.58	0.88	0.96	0.96
HIF1AN	0.96	0.63	0.38	0.99	0.12	0.39	0.32	0.78	0.45	0.60	0.74	0.85	0.55	0.81	0.30	0.88	0.99	0.73
HSPBAP1	0.96	0.54	0.53	0.99	0.75	0.84	0.44	0.83	0.96	0.49	0.74	0.22	0.91	0.50	0.23	0.88	0.94	0.71
JARID2	0.97	0.87	0.86	0.99	0.79	0.75	0.76	0.99	0.85	0.88	0.84	0.77	0.21	0.40	0.48	1.00	0.94	0.96
KDM1A	0.96	0.54	0.60	0.99	0.44	0.40	0.37	0.87	0.96	0.92	0.84	0.33	0.55	0.97	**2E-4**	0.88	0.96	0.90
KDM2A	0.96	0.69	0.86	0.99	0.74	0.59	0.73	0.87	0.74	0.75	0.74	0.97	0.75	0.72	0.71	0.91	0.96	0.96
KDM2B	0.96	0.89	0.28	0.99	0.23	0.75	0.44	0.88	0.71	0.76	0.74	0.97	0.91	0.87	0.66	0.88	0.94	0.47
KDM3A	0.96	0.38	0.81	0.99	0.95	0.88	0.97	0.88	0.93	0.46	0.74	0.51	0.91	0.97	0.09	0.70	0.96	0.22
KDM3B	0.98	0.94	0.06	0.99	0.31	0.19	0.37	0.73	0.71	0.72	0.74	0.34	0.80	0.64	0.95	0.88	0.96	0.65
KDM4A	0.98	0.65	0.92	0.99	0.75	0.79	0.73	0.98	0.45	0.60	0.74	0.52	0.55	0.81	0.57	0.88	0.95	0.63
KDM4B	0.96	0.38	**9E-5**	0.99	**1E-4**	**5E-5**	**1E-4**	**0.020**	0.45	0.66	0.81	**3E-4**	0.55	0.18	**0.009**	0.70	0.94	0.05
KDM4C	0.96	0.94	0.90	0.99	0.58	0.64	0.70	0.57	0.71	0.60	0.74	0.41	0.80	0.93	0.19	0.66	0.95	0.63
KDM5A	0.98	0.63	0.55	0.99	0.76	0.84	0.70	0.88	0.93	0.76	0.74	0.52	0.99	0.82	0.26	0.87	0.94	0.63
KDM5C	0.96	0.38	0.47	0.99	0.44	0.88	0.98	0.87	0.63	0.74	0.80	0.84	0.63	0.34	0.65	0.70	0.94	0.47
KDM6A	0.96	0.94	0.90	0.99	0.69	0.70	0.53	0.69	0.45	0.60	0.74	0.36	0.89	0.40	0.65	0.70	0.96	0.63
KDM6B	0.96	0.40	**0.019**	0.99	0.22	0.09	0.13	0.40	0.93	0.92	0.74	0.14	0.55	0.37	0.13	0.88	0.96	0.73
MBD1	0.98	0.63	0.87	0.99	0.75	0.86	0.80	0.99	0.93	0.66	0.74	0.84	0.95	0.93	0.69	0.66	0.94	0.71
MECP2	0.96	0.40	0.52	0.99	0.54	0.59	0.56	0.96	0.93	0.60	0.84	0.52	0.54	0.81	0.60	0.88	0.94	0.63
MLL	0.97	0.89	0.32	0.99	0.79	0.88	0.90	0.87	0.89	0.88	0.74	0.44	0.87	0.40	0.66	0.88	0.94	0.77
MLLT1	0.96	0.69	**0.019**	0.99	0.34	0.37	0.34	0.99	0.57	0.66	0.80	0.41	0.95	0.37	0.15	1.00	0.94	0.63
MLLT6	0.96	0.65	0.52	0.99	0.06	0.10	0.44	0.58	0.63	0.76	0.74	0.94	0.77	0.97	0.08	0.88	0.94	0.73
MYST1	0.96	0.54	**0.008**	0.99	0.15	**0.035**	0.09	0.25	0.37	0.75	0.74	**0.003**	0.21	0.50	0.08	0.88	0.94	0.72
MYST2	0.96	0.72	0.36	0.99	0.28	0.38	0.89	0.93	0.10	0.57	0.74	**0.043**	0.55	0.76	0.96	1.00	0.96	0.47
NSD1	0.96	0.63	0.87	0.99	0.31	0.40	0.84	0.78	0.45	0.60	0.74	0.81	0.81	0.87	0.56	0.88	0.94	0.63
PADI4	0.96	0.92	0.10	0.99	0.67	0.37	0.15	0.20	0.93	0.74	0.87	0.28	0.54	0.81	0.12	0.87	0.96	0.96
PCGF2	0.96	0.38	**0.008**	0.99	**0.003**	**0.029**	0.06	0.30	0.10	0.66	0.80	0.08	0.21	0.20	**0.046**	0.88	0.96	0.63
PCGF3	0.96	0.40	**0.014**	0.99	0.06	**0.025**	0.16	0.32	0.62	0.90	0.80	0.07	0.95	0.14	0.21	0.88	0.94	0.63
PHF8	0.96	0.96	0.55	0.99	0.56	0.51	0.84	0.99	0.46	0.74	0.84	0.82	0.96	0.86	0.56	0.91	0.94	0.87
PRMT1	0.96	0.38	0.87	0.99	0.79	0.51	0.70	0.40	0.93	0.74	0.74	0.62	0.55	0.94	0.06	1.00	0.96	0.92
PRMT5	0.96	0.89	0.60	0.99	0.59	0.82	0.73	0.99	0.74	0.74	0.74	0.63	0.55	0.97	0.30	0.70	0.96	0.96
PRMT6	0.98	0.59	0.90	0.99	0.56	0.82	0.91	0.93	0.89	0.60	0.74	0.97	0.55	0.80	0.78	1.00	0.94	0.80
PRMT8	0.96	0.97	**0.019**	0.60	**0.005**	**9E-4**	**0.020**	**0.016**	**0.047**	0.46	0.74	**4E-4**	0.54	0.17	0.28	0.88	0.94	0.47
SAP18	0.96	0.38	0.57	0.99	0.36	0.80	0.58	0.40	0.95	0.66	0.80	0.84	1.00	0.87	0.82	0.88	0.94	0.87
SAP30	0.96	0.89	0.25	0.99	0.69	0.73	0.64	0.73	0.63	0.57	0.74	0.11	0.99	0.72	**0.015**	1.00	0.96	0.87
SETD2	0.96	0.38	0.69	0.99	0.57	0.46	0.70	0.93	0.63	0.60	0.74	0.84	0.72	0.81	0.96	0.88	0.96	0.63
SETDB1	0.97	0.94	0.66	0.99	0.57	0.67	0.53	0.68	0.95	0.67	0.74	0.18	0.25	0.72	0.06	0.88	0.96	0.96
SIN3A	0.97	0.54	**0.008**	0.99	**0.009**	**0.009**	0.15	0.14	**0.047**	0.66	0.98	**0.032**	0.78	0.66	0.23	0.92	0.94	0.72
SIRT2	0.96	0.78	0.42	0.99	0.97	0.75	0.83	0.87	0.71	0.66	0.74	0.85	0.55	0.81	0.18	0.67	0.96	0.87
SMARCA4	0.98	0.63	0.55	0.74	0.79	0.46	0.64	0.32	0.80	0.76	0.74	0.52	0.63	0.72	0.06	0.88	0.94	0.71
SMARCAL1	0.96	0.94	0.13	0.99	0.34	0.64	0.70	0.87	0.93	0.75	0.89	0.52	0.95	0.23	0.55	0.88	0.96	0.63
SMARCE1	0.96	0.19	0.60	0.99	0.75	0.84	0.73	0.93	0.34	0.74	0.74	0.18	0.55	0.40	0.82	0.66	0.94	0.73
SUV39H1	0.98	0.69	**0.008**	0.99	**0.043**	**0.020**	**0.004**	0.14	0.45	0.60	0.74	**0.002**	1.00	0.81	**0.048**	0.70	0.96	0.96
SUV39H2	0.96	0.63	**1E-7**	0.99	**6E-6**	**9E-5**	**1E-4**	**0.005**	0.45	0.60	0.74	**6E-8**	0.63	**0.003**	**9E-8**	0.70	0.96	0.63

Supplementary Figure 3. *Association of gene expression levels with all known clinical variables in the UTSW cohort*. Clinical variables are shown across the top and genes across the vertical axis. Correlation values were derived as described in Methods and corrected for FDR. Significant FDR *q* values are highlighted. Positive correlations are depicted in red and negative correlations in blue font. Panel **A** shows associations in tumor samples (*n*=103) and panel **B** shows lack of these significant correlations in benign samples (*n*=83) after FDR correction.

S3B

UTSW Benigns

FDR *q*

									HER2									
	**Age**	**Race**	**Grade**	**Stage**	**ER**	**ER**	**PR**	**PR**	**IHC**	**FISH**	**HER2**	**TN**	**Ploidy**	**p53**	**Ki67**	**PrevTx**	**PostTx**	**Status**
Gene / Test	Corr.	Fisher	Corr.	Corr.	Corr.	*t* Test	Corr.	*t* Test	Corr.	Corr.	*t* Test	*t* Test	Fisher	*t* Test	Corr.	*t* Test	*t* Test	*t* Test
ARID1A	0.60	0.97	1.00	0.31	0.54	0.93	0.74	0.84	0.98	0.99	0.93	0.96	1.00	0.89	0.98	1.00	1.00	0.90
AURKB	0.77	0.97	1.00	0.74	0.69	0.72	0.97	0.98	0.98	0.99	0.95	0.96	1.00	0.94	0.98	1.00	1.00	0.96
CARM1	0.78	1.00	1.00	0.46	0.59	0.72	0.77	0.84	0.98	0.99	0.93	0.96	1.00	0.69	0.98	1.00	1.00	0.96
CHD3	0.61	0.97	1.00	0.14	0.81	0.83	0.77	0.87	0.98	0.99	0.93	0.94	1.00	0.69	0.98	1.00	1.00	0.96
DNMT3B	0.75	0.97	1.00	0.51	0.58	0.93	0.82	0.87	0.98	0.99	0.93	0.96	1.00	0.94	0.98	1.00	1.00	0.80
DOT1L	0.61	1.00	0.44	0.91	0.79	0.83	0.85	0.87	0.98	0.99	0.93	0.72	1.00	0.69	0.98	1.00	1.00	0.90
EHMT1	0.44	0.97	1.00	0.14	0.54	0.93	0.77	0.84	0.98	0.99	0.93	0.96	1.00	0.89	0.98	1.00	0.96	0.99
EHMT2	0.51	0.97	1.00	0.50	0.79	0.88	0.78	0.94	0.98	1.00	0.93	0.96	1.00	0.84	0.98	1.00	1.00	0.96
EZH2	0.44	0.97	1.00	0.98	0.97	0.83	0.80	0.87	0.99	0.99	0.93	0.98	1.00	0.89	0.98	1.00	1.00	0.96
HDAC10	0.49	0.97	1.00	0.46	0.40	0.72	0.70	0.84	0.98	0.99	0.93	0.94	1.00	0.94	0.98	1.00	1.00	0.90
HDAC3	0.44	0.97	1.00	0.70	0.62	0.72	0.70	0.84	0.98	0.99	0.93	0.99	1.00	0.69	0.98	1.00	1.00	0.96
HDAC4	0.48	0.97	1.00	0.50	0.54	0.93	0.70	0.84	0.98	0.99	1.00	0.94	1.00	0.86	0.98	1.00	1.00	0.96
HDAC9	0.91	1.00	0.44	0.46	0.56	0.72	0.70	0.84	0.98	0.99	0.93	0.51	1.00	0.94	0.98	1.00	1.00	0.96
HIF1AN	0.44	0.97	1.00	0.46	0.54	0.93	0.70	0.87	0.98	0.99	0.93	0.94	1.00	0.84	0.98	1.00	1.00	0.63
HSPBAP1	0.78	0.97	1.00	0.51	0.78	0.99	0.70	0.87	0.98	0.99	0.93	0.82	1.00	0.84	0.98	1.00	1.00	0.90
JARID2	0.44	1.00	1.00	0.73	0.78	0.92	0.80	0.88	0.98	0.99	0.93	0.94	1.00	0.89	0.98	1.00	1.00	0.90
KDM1A	0.44	0.97	1.00	0.33	0.78	0.99	0.77	0.87	0.98	0.99	0.93	0.94	1.00	0.69	0.98	1.00	1.00	0.96
KDM2A	0.61	0.97	1.00	0.68	0.69	0.99	0.70	0.84	0.98	0.99	0.93	0.94	1.00	0.94	0.98	1.00	1.00	0.96
KDM2B	0.54	1.00	1.00	0.17	0.54	0.72	0.70	0.84	0.98	0.99	0.93	0.94	1.00	0.89	0.98	1.00	1.00	0.99
KDM3A	0.44	0.97	1.00	0.14	0.78	0.92	0.88	0.98	0.98	0.99	0.95	0.94	1.00	0.89	0.98	1.00	1.00	0.80
KDM3B	0.44	0.97	1.00	0.14	0.97	0.99	0.77	0.88	0.98	0.99	0.93	0.98	1.00	0.69	0.98	1.00	1.00	0.90
KDM4A	0.77	0.97	1.00	0.14	0.56	0.72	0.80	0.84	0.98	1.00	0.93	0.94	1.00	0.84	0.98	1.00	1.00	0.63
KDM4B	0.44	0.97	1.00	0.11	0.97	0.99	0.77	0.88	0.98	0.99	0.93	0.98	1.00	0.94	0.98	1.00	1.00	0.96
KDM4C	0.44	0.97	1.00	0.46	0.97	0.99	0.80	0.98	0.98	0.99	0.93	0.94	1.00	0.84	0.98	1.00	1.00	0.96
KDM5A	0.60	0.97	1.00	0.21	0.57	0.72	0.85	0.87	0.98	1.00	0.96	0.51	1.00	0.97	0.98	1.00	1.00	0.99
KDM5C	0.61	1.00	1.00	0.14	0.48	0.99	0.85	0.87	0.98	0.99	0.93	0.96	1.00	0.89	0.98	1.00	0.96	0.35
KDM6A	0.44	0.97	1.00	0.91	0.97	0.99	0.85	0.87	0.98	0.99	0.93	0.94	1.00	0.89	0.98	1.00	1.00	0.90
KDM6B	0.91	1.00	1.00	0.29	0.54	0.72	0.82	0.96	0.98	0.99	0.93	0.94	1.00	0.71	0.98	1.00	1.00	0.96
MBD1	0.74	0.97	1.00	0.46	0.97	0.93	0.88	0.87	0.98	1.00	0.93	0.96	1.00	0.86	0.98	1.00	1.00	0.96
MECP2	0.73	0.97	1.00	0.36	0.78	0.92	0.77	0.87	0.98	0.99	0.95	0.94	1.00	0.94	0.98	1.00	1.00	0.63
MLL	0.44	0.97	1.00	0.29	0.78	0.72	0.88	0.87	0.98	0.99	0.93	0.94	1.00	0.69	0.98	1.00	0.96	0.96
MLLT1	0.68	0.97	1.00	0.46	0.54	0.99	0.77	0.84	0.98	0.99	0.93	0.94	1.00	0.99	0.98	1.00	1.00	0.44
MLLT6	0.44	1.00	1.00	0.17	0.48	0.73	0.77	0.84	0.98	1.00	0.93	0.94	1.00	0.94	0.98	1.00	1.00	0.80
MYST1	0.44	0.97	1.00	0.46	0.54	0.99	0.88	0.93	0.98	0.99	0.93	0.96	1.00	0.86	0.98	1.00	0.96	0.63
MYST2	0.44	0.97	1.00	0.14	0.97	0.99	0.77	0.88	0.98	0.99	0.93	0.98	1.00	0.69	0.98	1.00	1.00	0.96
NSD1	0.44	0.97	1.00	0.50	0.40	0.83	0.70	0.84	0.98	0.99	0.93	0.96	1.00	0.69	0.98	1.00	0.96	0.63
PADI4	0.77	0.97	1.00	0.56	0.78	0.92	0.74	0.87	0.98	0.99	0.93	0.98	1.00	0.69	0.98	1.00	1.00	0.63
PCGF2	0.78	1.00	1.00	0.73	0.78	0.99	0.81	0.84	0.98	0.99	0.93	0.94	1.00	0.88	0.98	1.00	1.00	0.80
PCGF3	0.48	1.00	1.00	0.31	0.23	0.72	0.70	0.84	0.98	0.99	0.95	0.90	1.00	0.69	0.98	1.00	1.00	0.35
PHF8	0.60	0.97	0.46	0.46	0.81	0.92	0.88	0.88	0.98	0.99	0.93	0.98	1.00	0.96	0.98	1.00	1.00	0.35
PRMT1	0.44	0.97	1.00	0.21	0.78	0.99	0.77	0.87	0.98	0.99	0.93	0.94	1.00	0.69	0.98	1.00	1.00	0.96
PRMT5	0.44	0.97	1.00	0.46	0.97	0.83	0.85	0.98	0.98	0.99	0.96	0.98	1.00	0.86	0.98	1.00	1.00	0.96
PRMT6	0.78	0.97	0.46	0.46	0.78	0.99	0.82	0.84	0.98	0.99	0.93	0.97	1.00	0.89	0.98	1.00	1.00	0.63
PRMT8	0.78	0.97	1.00	0.33	0.78	0.83	0.97	0.84	0.98	1.00	0.93	0.98	1.00	0.99	0.98	1.00	1.00	0.96
SAP18	0.77	0.97	1.00	0.17	0.78	0.99	0.85	0.88	0.98	0.99	0.93	0.94	1.00	0.97	0.98	1.00	1.00	0.80
SAP30	0.44	0.97	0.97	0.91	0.97	0.93	0.80	0.87	0.98	0.99	0.93	0.96	1.00	0.89	0.98	1.00	1.00	0.63
SETD2	0.44	0.97	0.97	0.17	0.78	0.88	0.82	0.87	0.98	1.00	0.96	0.94	1.00	0.89	0.98	1.00	1.00	0.96
SETDB1	0.44	0.97	1.00	0.46	0.97	0.99	0.82	0.88	0.98	0.99	0.95	0.94	1.00	0.94	0.98	1.00	1.00	0.63
SIN3A	0.51	0.97	1.00	0.78	0.40	0.72	0.70	0.84	0.98	0.99	0.93	0.94	1.00	0.89	0.98	1.00	0.96	0.63
SIRT2	0.62	1.00	0.51	0.64	0.78	0.83	0.82	0.87	0.98	1.00	0.96	0.94	1.00	0.84	0.98	1.00	1.00	0.96
SMARCA4	0.44	0.97	0.51	0.31	0.23	0.72	0.77	0.84	0.98	0.99	0.93	0.51	1.00	0.86	0.96	1.00	1.00	0.96
SMARCAL1	0.44	0.97	0.51	0.14	0.78	0.83	0.74	0.84	0.98	0.99	0.93	0.94	1.00	0.69	0.98	1.00	1.00	0.96
SMARCE1	0.61	0.97	1.00	0.46	0.81	0.72	0.77	0.87	0.98	0.99	0.93	0.94	1.00	0.84	0.98	1.00	1.00	0.96
SUV39H1	0.73	1.00	1.00	0.33	0.79	0.99	0.77	0.88	0.98	0.99	0.93	0.94	1.00	0.94	0.98	1.00	1.00	0.96
SUV39H2	0.73	0.97	1.00	0.58	0.78	0.99	0.77	0.87	0.98	0.99	0.95	0.94	1.00	0.89	0.98	1.00	1.00	0.96

A

FDR *q* METABRIC Dataset (*n* = 1,992)

**Gene** / Test	**Grade** Correl.	**Size** Correl.	**Stage** Correl.	**ER** *t* Test	**PR** *t* Test	**HER2** *t* Test	**TN** *t* Test
AURKB	2E-118	4E-13	0.84	3E-50	6E-44	6E-7	1E-38
DNMT3B	2E-56	3E-8	0.004	2E-36	4E-41	4E-22	2E-13
EZH2	7E-93	3E-12	0.52	4E-46	2E-38	5E-7	1E-32
SUV39H1	5E-58	1E-7	0.60	1E-21	7E-20	3E-8	4E-14
SUV39H2	3E-9	1E-5	4E-5	2E-6	4E-7	0.16	4E-4
CHD3	0.82	0.07	3E-7	0.11	0.56	0.14	0.81
KDM4B	1E-37	0.16	2E-15	2E-136	2E-51	1E-26	2E-106
MYST1	4E-29	0.96	1E-7	8E-35	4E-27	4E-20	4E-17
PCGF2	5E-14	0.48	5E-6	0.80	0.048	1E-11	5E-45
PCGF3	0.39	0.50	0.60	0.021	0.048	0.039	0.43
PRMT8	0.73	0.96	0.60	0.003	0.001	0.012	0.030
SIN3A	6E-28	0.16	3E-10	4E-50	4E-25	1E-5	5E-35

B

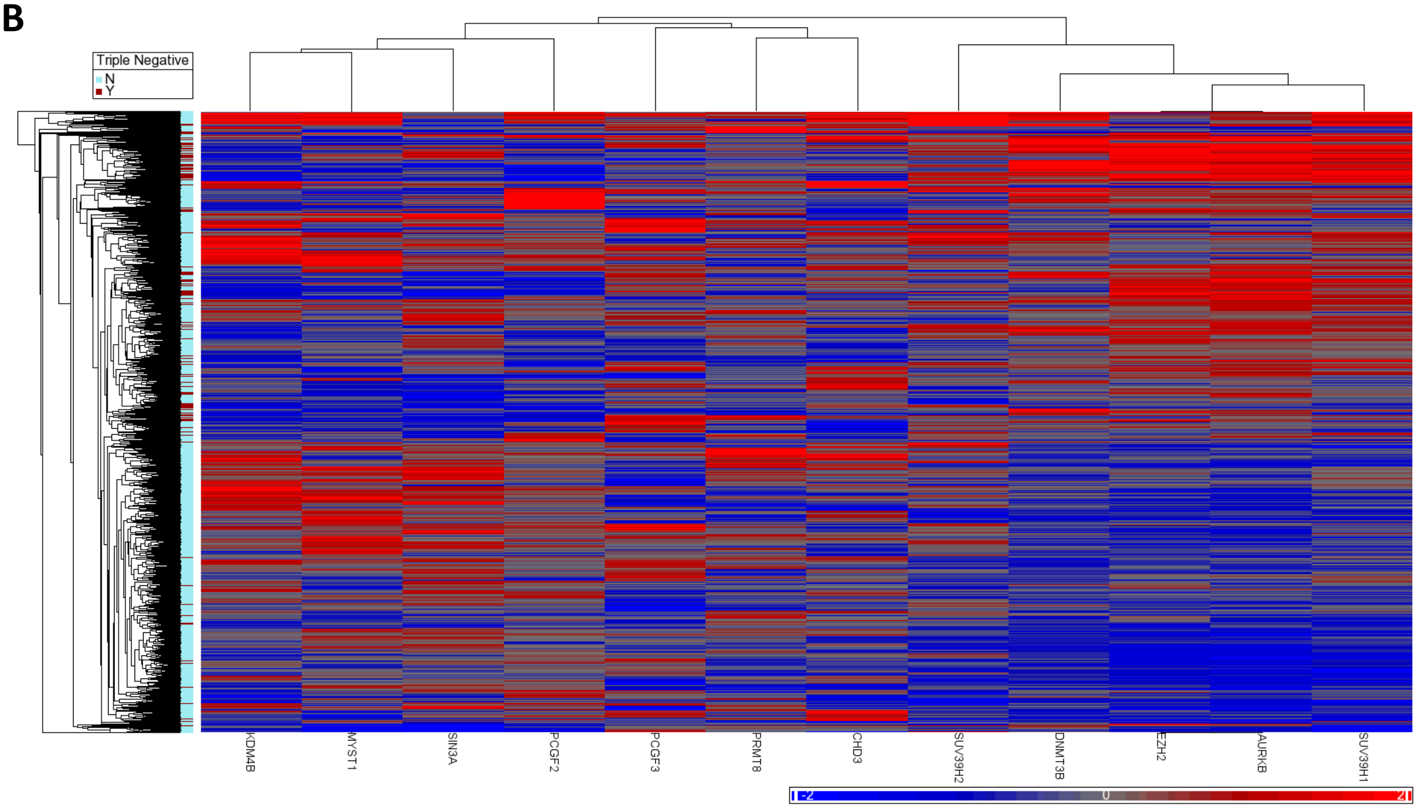

Supplementary Figure 4. *The Epigenetic Signature is also validated in the METABRIC dataset and clusters better than random genes*. **A**, FDR-corrected *p* values (*q*) are shown for the twelve genes of the Epigenetic Signature in the METABRIC dataset (*n*=1992). Positive correlations are shown in red and negative correlations in blue. Note that 10/12 UTSW epigenetic genes correlate with Triple Negative (TN) status and 9/12 with tumor grade. **B**, Heatmap and unsupervised hierarchical clustering of tumors samples of the METABRIC cohort based on the gene expression of the Epigenetic Signature. **C**, Unsupervised hierarchical clustering was performed on the TCGA dataset according to the levels of expression of the eleven genes of the Epigenetic Signature or three different sets of eleven genes randomly chosen from 20,534 genes/transcripts. Arbitrary distances were calculated between the main cluster of normal samples (present in all cases) and the remaining samples. A one-sample *t*-test was performed to compare the distance of the eleven epigenetic genes vs. the other sets of random genes. Histological information, PAM50 status and TN status are shown in the color coded side bars, for reference. **D**, Unsupervised hierarchical clustering of eleven genes randomly chosen from the 55 epigenetic genes excluding the twelve genes of the Epigenetic Signature. (See next page for panel **C**).

S4C

11 Epigenetic genes

d = 0.0
P = 0.044

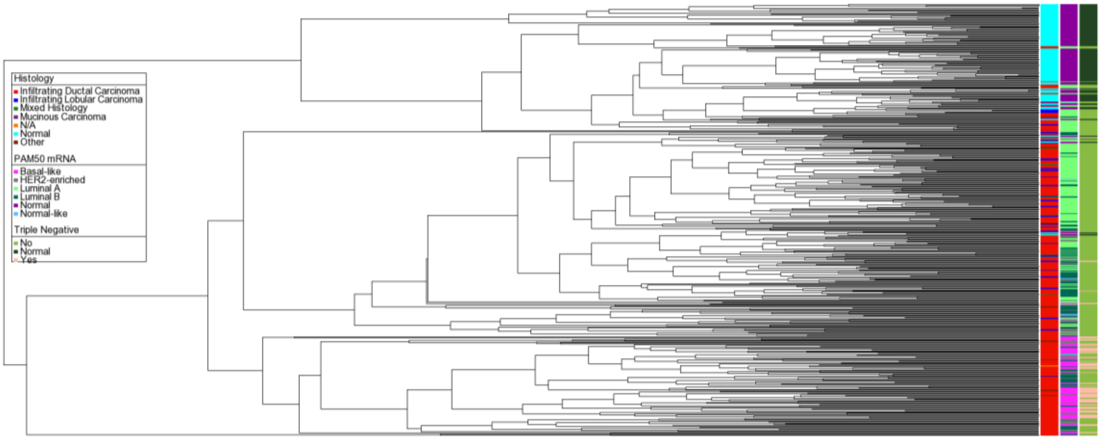

11 Random genes

d = 85.2

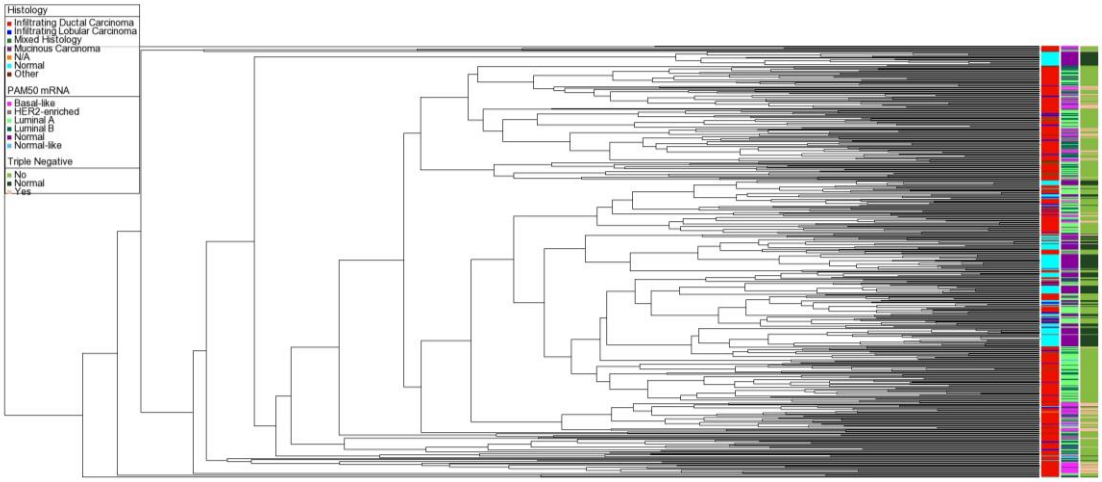

11 Random genes

d = 134.5

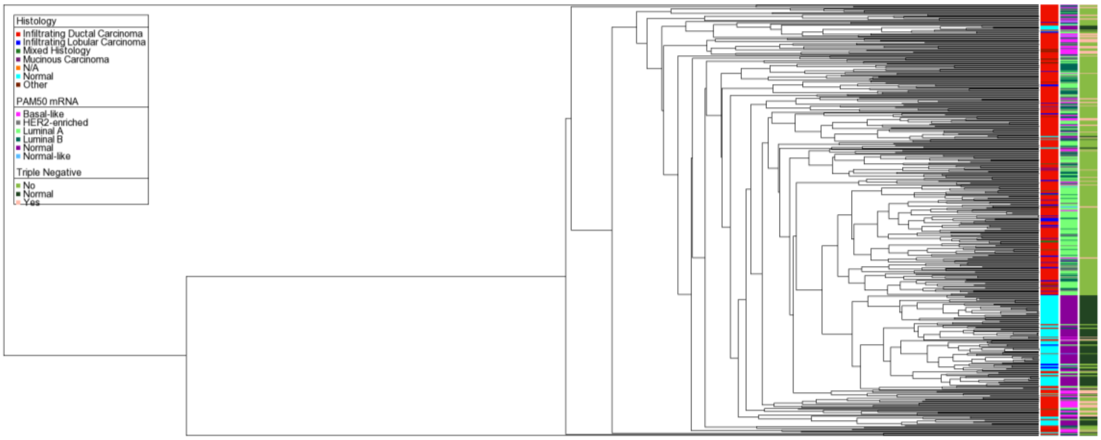

11 Random genes

d = 65.0

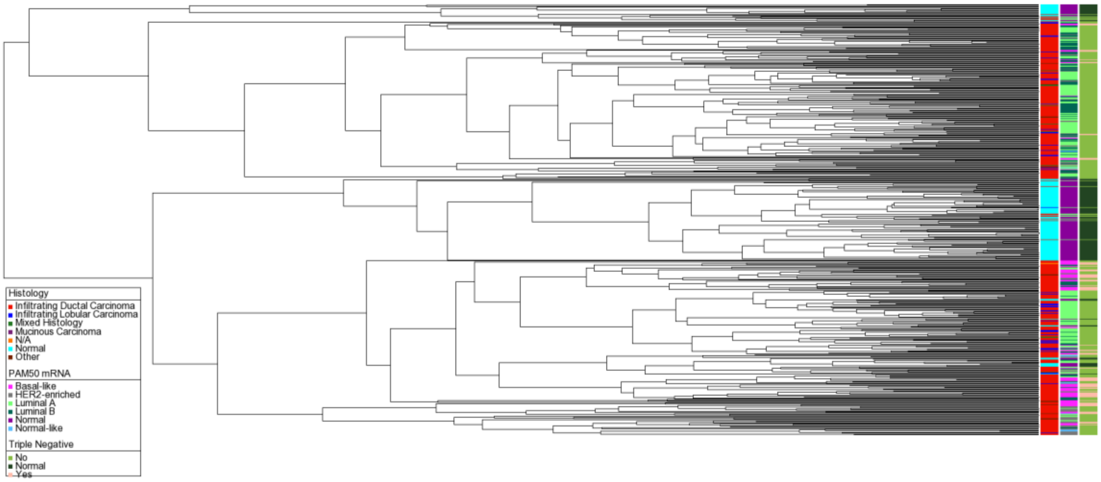

S4D

11 Random Epigenetic genes

d = 88.9

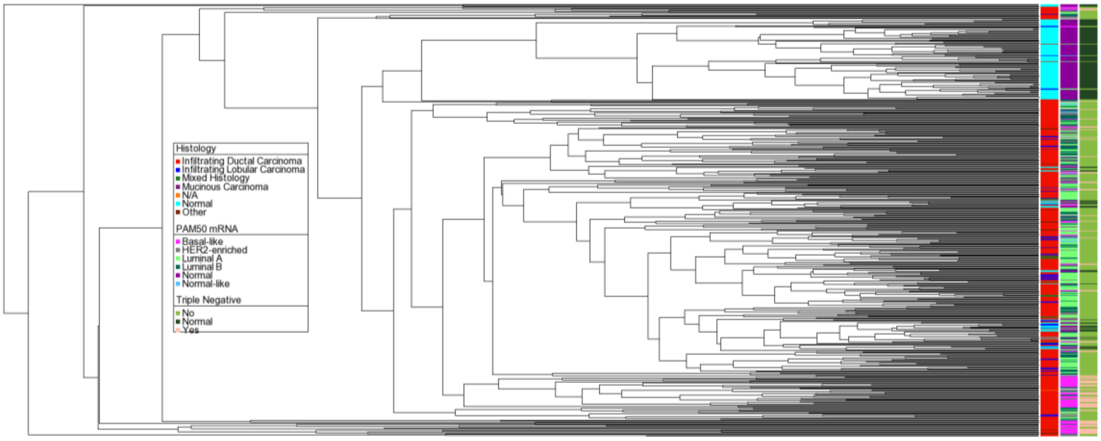

11 Random Epigenetic genes

d = 112.4

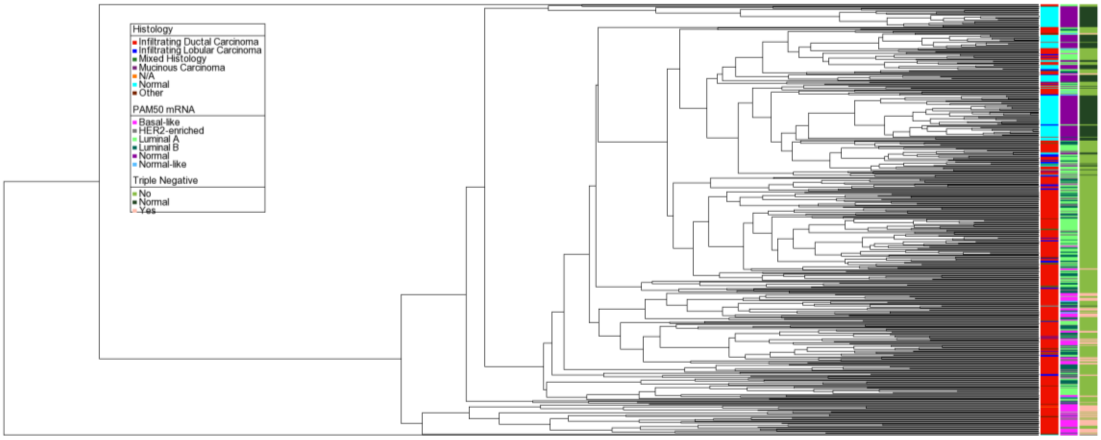

11 Random Epigenetic genes

d = 104.3

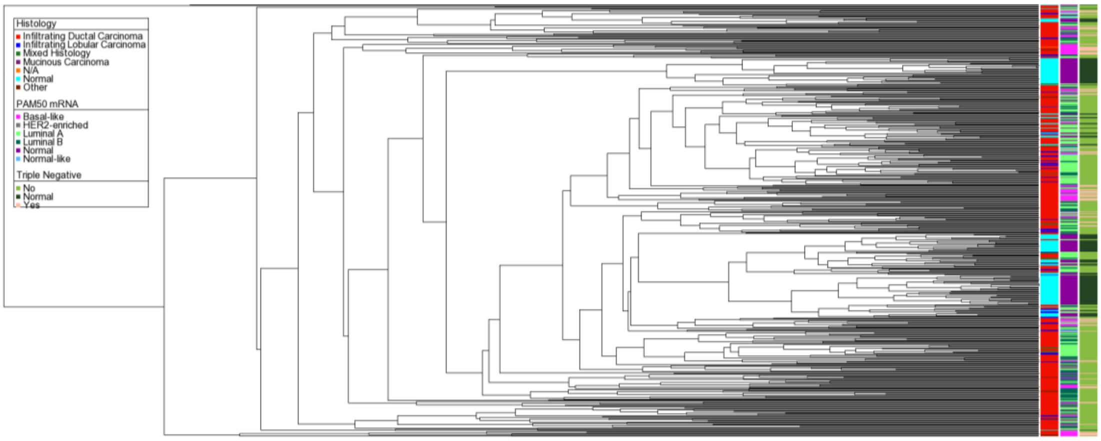

11 Random Epigenetic genes

d = 113.6

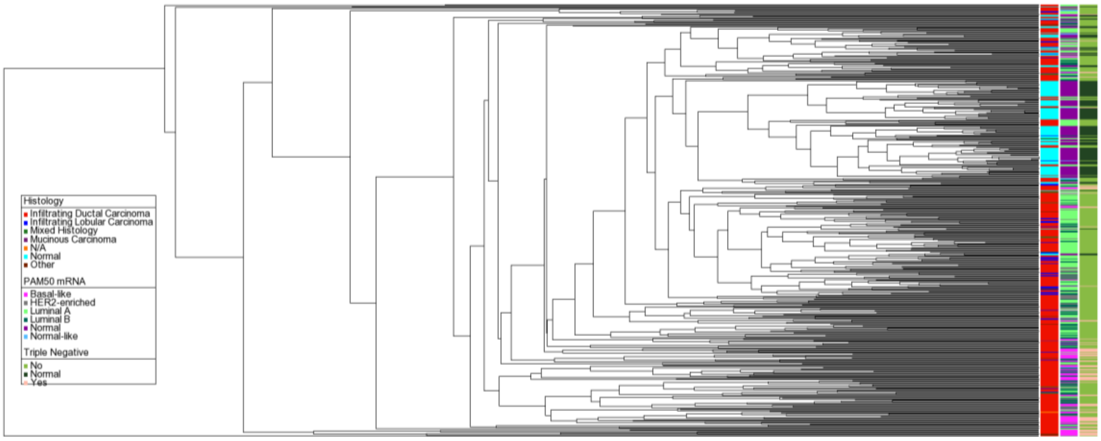

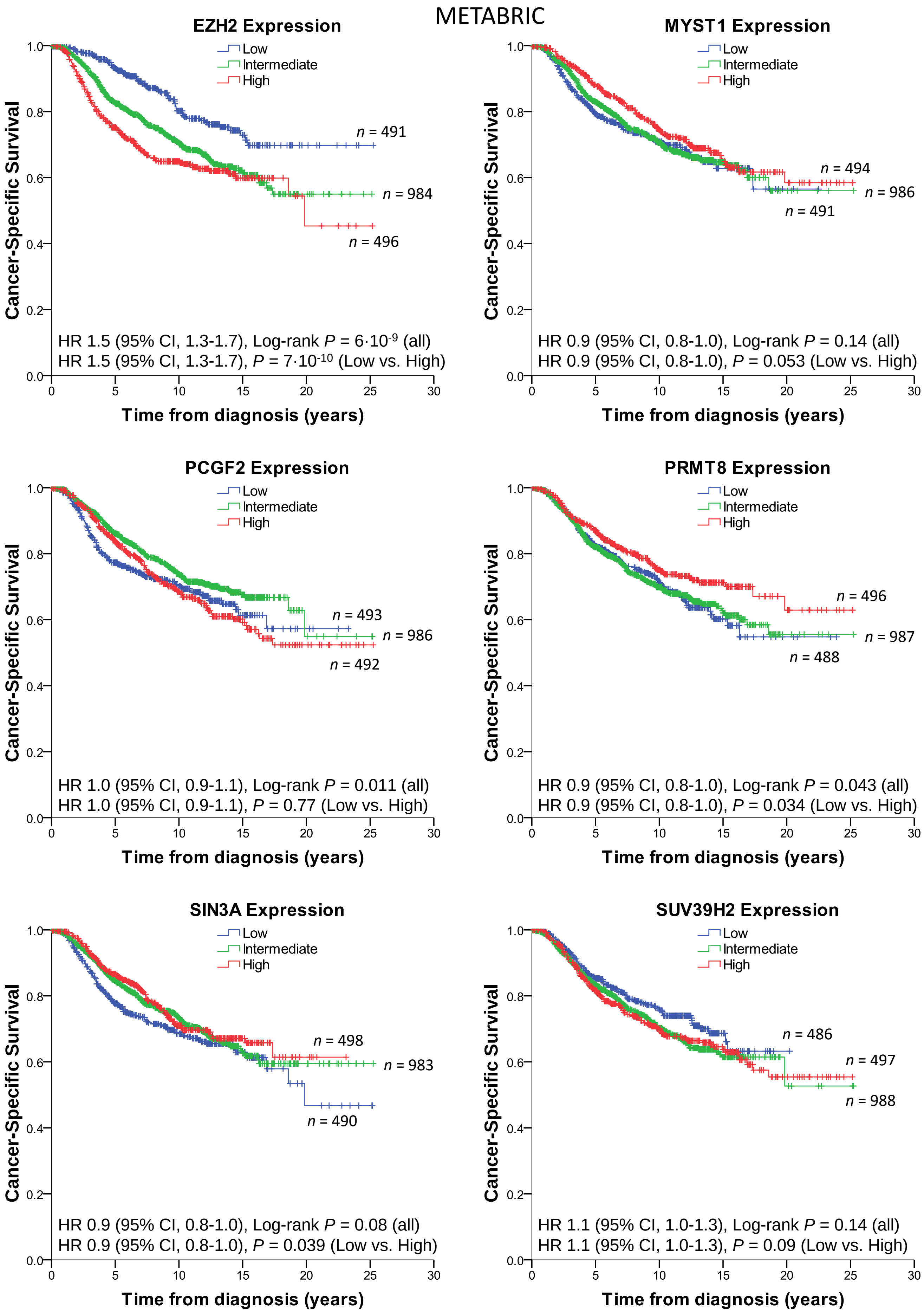

Supplementary Figure 5. *Kaplan-Meier survival curves as determined by expression level quartiles of epigenetic genes in the METABRIC dataset.* Blue, 1st quartile; Green, 2nd and 3rd quartiles; Red, 4th quartile. Censored data are designated by crosses.

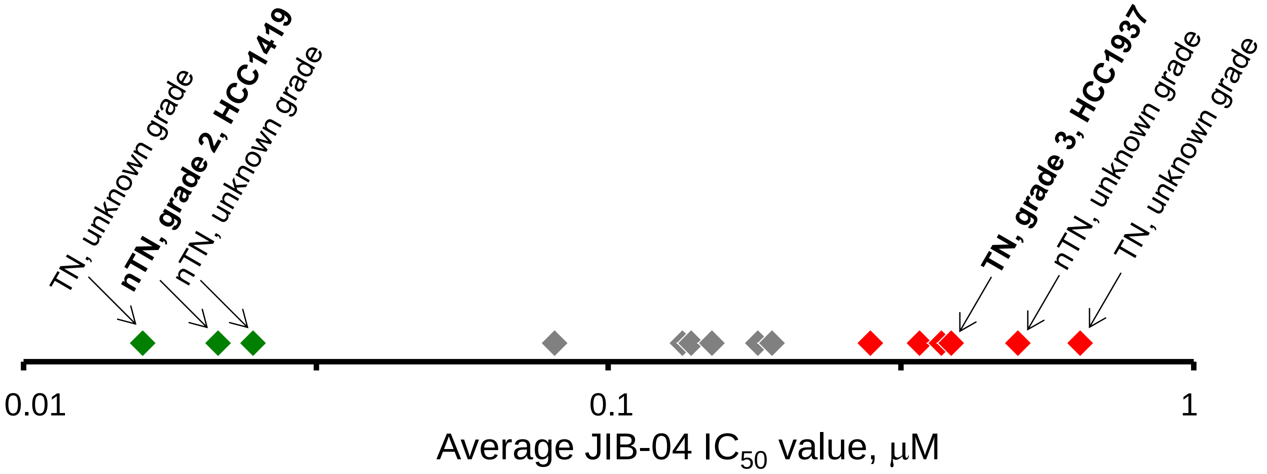

Supplementary Figure 6. *Response of BCa cell lines to Jumonji inhibition by TN status and grade, when known.* IC_{50} values in response to JIB-04 inhibition are shown for individual BCa cell lines (see Figure 6A). Each diamond represents a cell line IC_{50} value and TN and grade status are given, when known for the most sensitive and most resistant lines.

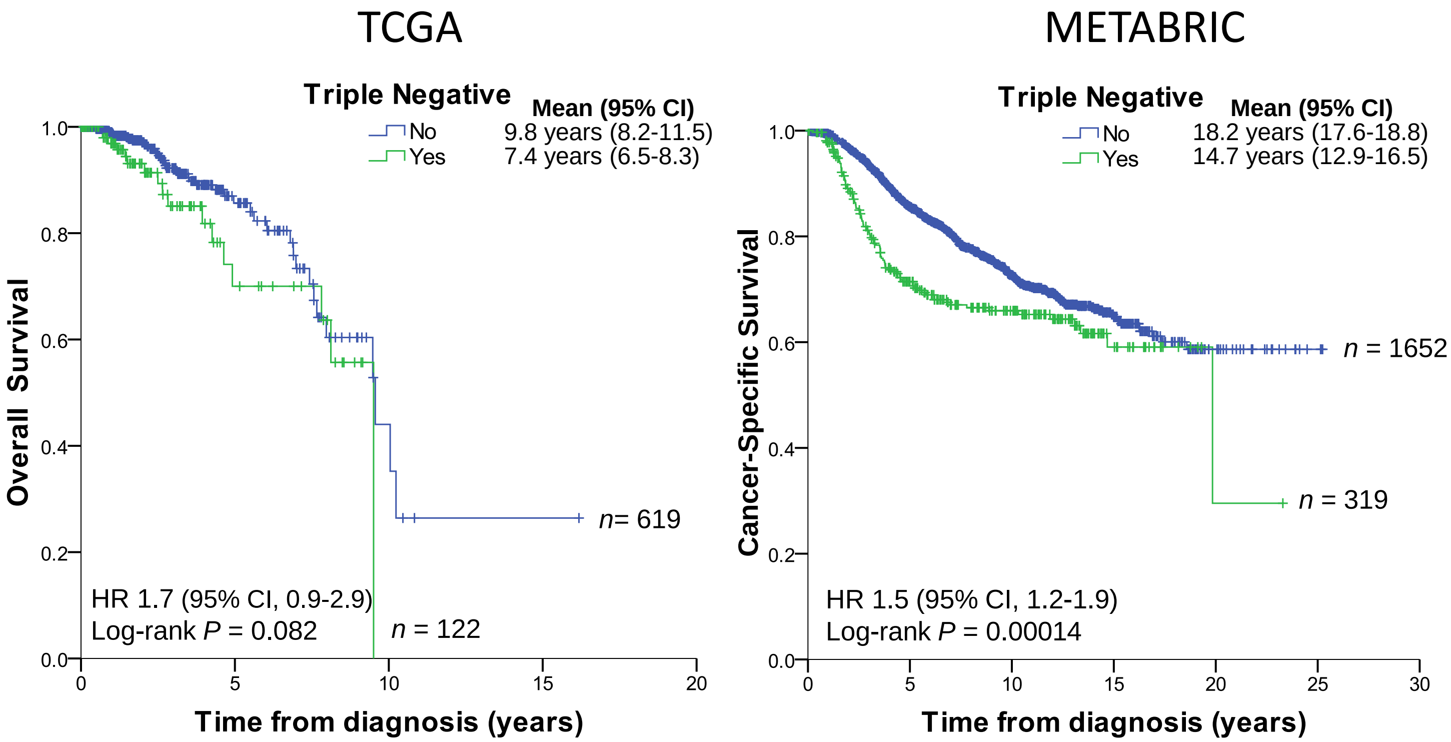

Supplementary Figure 7. *Triple negative status is associated with poor survival.* Kaplan-Meier survival curves of the TCGA and METABRIC datasets based on the triple negative status. Please note the lower significance compared to the Epigenetic Signature in Figure 6.
